# Graphene Oxide–Enamino-Xanthene Charge-Transfer Hybrids as High-Performance Sensitizing Interfaces for TiO_2_ Photoanodes

**DOI:** 10.1021/acsami.5c20186

**Published:** 2025-12-31

**Authors:** Carlos Martínez-Barón, Juan Manuel Garrido-Zoido, Miguel Á. Álvarez Sánchez, Pedro Cintas, Juan C. Palacios, Alejandro Ansón-Casaos, María Victoria Gil, Ana M. Benito, Wolfgang K. Maser

**Affiliations:** † 120031 Instituto de Carboquímica, ICB-CSIC, Zaragoza 50018, Spain; ‡ Departamento de Química Orgánica e Inorgánica, Facultad de Ciencias, and Instituto del Agua, Cambio Climático y Sostenibilidad (IACYS), 16759 Universidad de Extremadura, Badajoz 06006, Spain

**Keywords:** graphene oxide, xanthene dyes, charge-transfer hybrids, TiO_2_ photoanodes, photoelectrochemical interfaces

## Abstract

Hybrid materials that combine visible-light absorption with efficient charge separation across interfaces are essential for advancing photoelectrochemical (PEC) energy conversion technologies. In this study, we report the synthesis of charge-transfer hybrids composed of graphene oxide (GO) and enamino-xanthene (NH_2_-X) dyes, prepared via a simple and sustainable liquid-phase mixing approach. Spectroscopic analyses reveal strong interface interactions between GO and NH_2_-X, leading to suppressed fluorescence and altered electronic transitions, consistent with ground-state charge-transfer processes. When interfaced with TiO_2_ as a sensitizing layer, the resulting photoanodes deliver a 3.5-fold enhancement in photocurrent, faster saturation kinetics, and improved photopotential generation under visible-light illumination. Electrochemical impedance spectroscopy confirms reduced interface resistance and enhanced charge carrier mobility at both solid–solid and solid–liquid interfaces. The synergistic integration of GO and NH_2_-X establishes a high-performance sensitizing interface for TiO_2_ photoanodes, offering a scalable and environmentally benign route toward advanced PEC applications.

## Graphical abstract

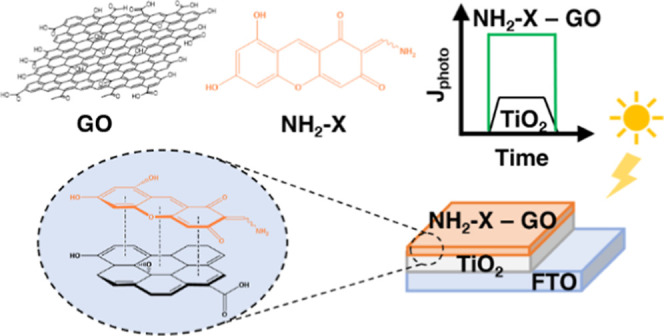

## Introduction

The development of efficient photoelectrochemical (PEC) systems for solar energy conversion heavily relies on materials that can extend light absorption into the visible range and facilitate rapid charge separation across interfaces.^1–4^ Titanium dioxide (TiO_2_), a semiconducting metal oxide commonly employed as a photoanode to this end, suffers from limited visible-light absorption, necessitating sensitization strategies to improve its PEC performance. In particular, nanocrystalline anatase TiO_2_, behaving as an intrinsically n-type semiconductor due to oxygen-vacancy-related donor states, offers, when processed into porous films of nanocrystalline morphology, abundant adsorption sites for sensitizing molecules, thus facilitating the establishment of intimate electronic dye/TiO_2_ interfacial contact.^5–8^ Organic dyes, particularly xanthene derivatives, have been extensively explored for this purpose due to their strong fluorescence and tunable photophysical properties.^9–12^ However, dye-sensitized photoanodes often face challenges related to poor charge-transfer efficiency and photodegradation, which hamper their practical applicability.^9,13^

To address these limitations, interface engineering using carbon nanomaterials has emerged as a promising approach. Graphene oxide (GO), with its rich surface chemistry and electronic versatility,^14,15^ enables the formation of hybrid materials with enhanced charge transport properties.^16–21^ GO has been successfully integrated with semiconducting oxides or conjugated polymers to improve the interface charge dynamics and device performance.^17–25^ These findings suggest that GO can serve as a functional interface modifier in dye-sensitized systems.

Recent advances in xanthene chemistry have led to the emergence of a new family of dyes, namely, enamino-xanthenes.^26^ These are sustainably synthesized via a catalyst-free, one-pot tandem protocol using 2,4,6-trihydroxybenzaldehyde and primary amines, with water as the sole byproduct, which is in line with the principles of green chemistry. Importantly, the resulting dyes feature peripheral substitution with aliphatic amines. This stands in contrast to conventional xanthene derivatives such as eosin Y and fluorescein,^27–29^ which are functionalized at the central ring of their tricyclic core structure. The peripheral functionalization of xanthene moieties thus introduces new possibilities for modulating their photochemical behavior, furnishing highly colored and fluorescent compounds.^26^ Moreover, the rigid π-conjugated framework and strong absorption of enamino-xanthenes in the visible range make them suitable candidates for hybrid sensitization with GO.

In this study, we explore the simplest member of this new xanthene family, referred to as NH_2_-X, and investigate its hybridization with GO as a strategy to overcome the limitations of conventional dye sensitization. To this end, we combine NH_2_-X with GO to form charge-transfer hybrids via a simple liquid-phase mixing process. We investigate their photophysical behavior and apply them as sensitizing layers on anatase TiO_2_ film photoanodes made from a widely employed commercial nanoparticulate anatase paste. Through a comprehensive set of PEC and impedance spectroscopy measurements, we demonstrate that these hybrids enhance photocurrent generation, accelerate charge separation, and reduce interfacial resistance. Our results highlight the functional role of NH_2_-X–GO interfaces in improving PEC performance and offer a scalable, environmentally benign route to advanced sensitizer design for TiO_2_ photoanodes.

## Experimental Methods

### Preparation of Enamino-Xanthene Dye

The synthetic procedure and subsequent characterization of the employed enamino-xanthene (2-(aminomethylene)-6,8-dihydroxy-1H-xanthene-1,3­(2H)-dione*,* in the following labeled NH_2_-X, whereby X denotes the hydrocarbon core C_14_H_7_O_5_) has been previously reported.^26^ In brief, to a solution of 2,4,6-trihydroxybenzaldehyde (3.0 mmol) in absolute ethanol (2 mL), a 25% aqueous ammonia solution (1.5 mmol) was slowly added. After a reaction time of 72 h at room temperature, a solid was formed, which was filtered, washed successively with cold ethanol, cold acetone, and ethyl ether, and dried on silica gel (yield 62%). All reagents (THB and ammonia) and solvents were purchased from commercial suppliers and used without further purification. The spectral characterization of NH_2_-X is detailed in the Supporting Information (Section S1).

### Preparation of Graphene Oxide

In a first step, graphite oxide was prepared using a modified Hummers method.^21,30^ Specifically, 5 g of graphite flakes were inserted into 170 mL of H_2_SO_4_ and 3.75 g of NaNO_3_, cooled by an ice bath. After the mixture was stirred for 30 min, 25 g of KMnO_4_ was slowly added. The reaction was kept at 0 °C for another 30 min. Subsequently, the ice bath was removed, the mixture was warmed up to 35–40 °C, and stirred overnight. The reaction was terminated by slowly adding 250 mL of deionized water and then 20 mL of H_2_O_2_ (30%) solution. The resulting dispersion was filtered, and the obtained powder material was repeatedly washed with 400 mL of HCl/H_2_O (1:10 v/v) to remove any metal ions followed by washing with deionized water until neutral pH was obtained. Drying the product at room temperature afforded graphite oxide. Subsequently, graphite oxide was dispersed in water at a concentration of 2 mg/mL. Upon sonication in an ultrasonic bath (working at 45 kHz as the nominal frequency) for 30 min, a brown dispersion of individual graphene oxide (GO) flakes was obtained. Characterization results of the freeze-dried solid GO powder product, including X-ray diffraction, Raman, Fourier transform infrared (FTIR), and thermogravimetric analysis, are detailed in the Supporting Information, Section S2.

### Graphene Oxide–Enamino-Xanthene Hybrids

Hybrids of graphene oxide (GO) and enamino-xanthene (NH_2_-X) were obtained by a solution mixing approach. Keeping the NH_2_-X concentration constant, the synthesis procedure covers the following steps (see also Figure 1) First, preparation of (i) a methanolic NH_2_-X solution at a concentration of 0.025 mg/mL and (ii) a methanolic xanthene solution of 0.025 mg/mL containing GO at a concentration of 0.2 mg/mL. Second, stepwise addition of controlled amounts of (ii) to (i), using 10 μL of (ii) and 2 mL of (i) for the photophysical characterization, or 20 mL of (i) for their use as a sensitizing layer in photoanodes, respectively. Third, a sonication process in an ice bath using an ultrasound probe (20 kHz) for a time of 15 min (60% amplitude, 0.5 cycles). Depending on the employed NH_2_-X volume, hybrids with GO loadings ranging from 4 to 40 wt % were used for photophysical analyses in solution, while modified photoanodes were prepared with hybrids covering GO loadings within a range of 0.4–40 wt %.

**Figure 1:**
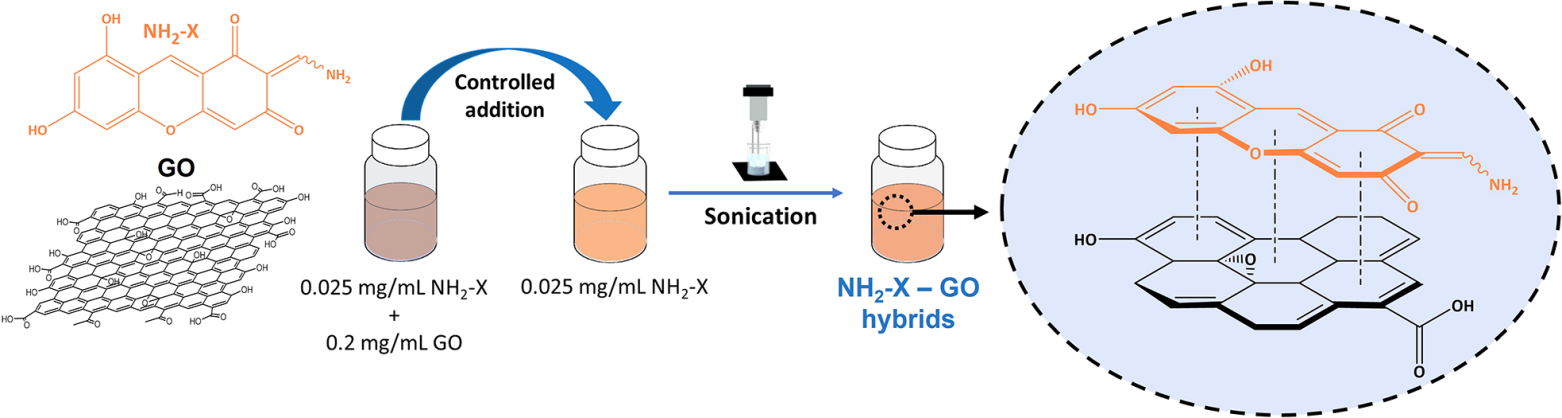
Chemical structure of the NH_2_-X dye molecule and preparation scheme of NH_2_-X–GO charge-transfer hybrids.

### Fabrication of TiO_2_ Film Photoanodes and Sensitization

Fluorinated tin oxide (FTO)-coated glass substrates (80 Ω/□, 80 nm thickness, 25 × 10 × 1.1 mm^3^, obtained from Solems S.A.) were cleaned by its immersion in ultrapure water with Hellmamex III detergent and ethanol for 15 min each under sonication. Calcination of the substrates at 500 °C in air for 30 min and an ozone treatment for 25 min was carried out. Subsequently, a commercial TiO_2_ paste (GreatCell Solar 18 NR-AO, composed of well-defined active anatase TiO_2_ nanoparticles) was screen-printed onto the cleaned FTO substrate forming a TiO_2_ film, whereby an amount of 0.6 mg covered a surface of 1 cm^2^. Next, the nanoparticulate TiO_2_ film was sintered in an oven following a standard heating protocol: 5 min at 325 °C, 5 min at 375 °C, 5 min at 450 °C, and 15 min at 500 °C to achieve a nanoparticulate anatase TiO_2_ film with a thickness of approximately 4.5 μm, as determined by profilometric measurements using a Dektak XT apparatus.

For TiO_2_ film sensitization, films were placed onto a hot plate kept at 40 °C and a 0.025 mg/mL methanolic solution of the employed xanthene was spray-coated over the TiO_2_ films using a semiautomatic spray coater (Nadetech ND-SP Ultrasonic PRO). The nozzle was placed 60 mm above the substrates, and a spraying flow rate of 30 mL/h at a scanning speed of 1000 mm/s was applied. A homogeneous coverage layer of TiO_2_ was achieved upon 40 spray coating steps, resulting in an overall deposited amount of dye of 0.06 mg/cm^2^. In the case of the hybrid GO/xanthene dispersions, the same sensitization procedure was employed.

### Photophysical Characterization

Photophysical measurements were carried out on samples in solutions (NH_2_-X and hybrid materials with increasing GO amounts at constant NH_2_-X concentration) placed in 10 mm path-length cuvettes. UV–vis spectra were acquired by using a dual-beam Shimadzu UV-2401PC spectrometer. Photoluminescence spectra were collected with a Horiba Jobin Yvon Fluoromax-P apparatus using emission and excitation slits of 1 mm. Photoluminescence lifetime measurements were carried out for a 10^–5^ M NH_2_-X methanolic solution in quartz cuvette on a Horiba Jobin-Yvon Fluorolog FL-3–11 apparatus equipped with a Fluoromax phosphorimeter accessory containing a Horiba Jobin Yvon light-emitting diode array with a pulse duration time below 1.2 ns.

### Structural and Morphological Characterization

Attenuated total reflection FTIR measurements were recorded on a PerkinElmer Spectrum 100 Fourier transform infrared spectrometer, and Raman spectra were acquired employing a micro-Raman LabRam HR800 UV spectrometer (Horiba Jobin Yvon) apparatus using an excitation wavelength of 532 nm and a spot size of 1 μm. Field emission scanning electron microscopy (FE-SEM) was performed using a Carl Zeiss MERTLINTM operating at a voltage of 5 kV and a working distance of 5.5 mm.

### PEC Characterization

PEC experiments were performed using an Autolab PGSTAT302 instrument from MetrOhm. The light source was a 150 W xenon lamp in a laboratory solar simulator from LOT-Oriel. A cold mirror was employed during measurements for partly removing the UV part of the solar spectrum while increasing the visible contribution (420–680 nm), thus having 25 mW/cm^2^ of incident power at the distance of the targeted electrode. The PEC cell consisted of a glass container with a quartz window and three electrodes, employing an Ag/AgCl as a reference electrode (3 M NaCl, *E*° = 0.210 V vs SHE) and a graphite bar as a counter electrode. Fabricated photoanodes were used as the working electrodes. The employed electrolyte was an aqueous 0.1 M solution of Na_2_SO_4_, previously purged with N_2_ during 10 min before the experiments. The cyclic voltammetry (CV) measurements were performed at 20 mV/s in the range of 1.1–0.4 V, starting at 0.4 V vs Ag/AgCl, both in dark and under illumination conditions. On/off transient photocurrent measurements were performed at 0 V (vs Ag/AgCl). PEC measurements were carried out in the following order: (i) CV in the dark and (ii) CV under illumination and transient photocurrent. Incident photon-to-current efficiency (IPCE) spectra were acquired by focusing monochromatized light, facilitated by a monochromator (LOT Oriel MSH-300) coupled to a solar simulator light source onto the photoanode. Irradiation intensity at each wavelength was acquired by using a photometer (Newport). Electrochemical impedance spectroscopy (EIS) experiments were carried out at an open circuit potential under dark and illumination conditions. In general, the electrochemical experiments were carried out with the assistance of sacrificial agent triethanolamine (TEOA^+^). To this end, TEOA was added to the electrolyte until a concentration of 0.1 M followed by a dropwise addition of nitric acid to vary the pH from 10 to 7. Preconditioning of NH_2_-X–GO hybrids prior to PEC characterization was carried out through a linear sweep voltammetry (LSV) step in 0.1 M Na_2_SO_4_ under dark conditions and after this process, TEOA^+^ was added.

## Results and Discussion

As mentioned in the introductory remarks, the extended π-conjugation along with its rigid chemical structure makes NH_2_-X a potential candidate for establishing strong interfacial interactions with planar GO sheets. The fabrication of NH_2_-X–GO charge-transfer hybrids was carried out through a solution mixing process whereby the NH_2_-X concentration was kept constant upon the controlled addition of GO (Figure 1).

The formation of the hybrids with different amounts of GO was systematically followed by photophysical characterization (Figure 2). The UV–vis spectrum of NH_2_-X (Figure 2a) reveals three intense bands with maxima located at 472 nm (2.63 eV), 315 nm (3.94 eV), and 204 nm (6.06 eV). These represent transitions corresponding to the first, second, and third electronic excited singlet states *S*_1_ (lowest intensity), *S*_2_ (intermediate intensity), and *S*_3_ (highest intensity), respectively. They predominantly arise from combinations of multiple π–π* transitions, typical for the tricyclic core structure of xanthenes with its delocalized π-conjugation.^31^ Weak contributions at 403 nm (3.08 eV) and 268 nm (4.63 eV) most likely involve nonbonding molecular orbitals of the attached functional groups.^29^ Each of the three major bands exhibits the distinctive vibronic structure of conjugated systems, whereby the low-energetic *S*_1_ band reveals the fundamental vibrational quanta *A*_00_ at 496 nm (2.50 eV), *A*_01_ at 472 nm (2.63 eV), and *A*_02_ at 443 nm (2.80 eV), with *A*_01_ exhibiting the strongest intensity (for more details on vibrational modes in NH_2_-X, see Supporting Information, Section S3). The energetically lowest-lying band with maximum at 472 nm corresponds to the *S*_0_–*S*_1_ π–π* HOMO–LUMO transition, being responsible of the strong fluorescence of NH_2_-X. The corresponding 2D excitation–emission fluorescence plot of NH_2_-X (Figure 2b) clearly exhibits one single emission center at 528 nm of highest intensity when excited with wavelengths falling into the S_1_ band (450–520 nm). Excitations at lower wavelengths in the range of the *S*_2_ and *S*_3_ electronic states also contribute to this emission. Hence, electrons excited from the ground state *S*_0_ of the NH_2_-X molecule to the *S*_2_ or *S*_3_ states relax nonradiatively via internal conversion to the *S*_1_ state from which they radiatively return to the *S*_0_ ground state. *S*_3_ and *S*_2_ with their highest absorption intensity thus provide major routes for populating the fluorescence-causing *S*_1_ state. Upon mixing with GO, no features specific to this carbon nanostructure are encountered in the NH_2_-X–GO spectra (see Supporting Information, Figures S4 and S5). This observation is the very first hint for the establishment of interactions between NH_2_-X and GO in the liquid phase. To analyze the influence of GO on the NH_2_-X spectra, the original NH_2_-X–GO spectra (Supporting Information, Figure S4) are corrected by the contribution of noninteracting GO (removal of GO background) and normalized to the *A*_01_ intensity maximum of the excited *S*_1_ state, as shown in Figure 2a. The most striking changes encountered relate to the *S*_3_ state. Indeed, its main contribution at 204 nm (*A*″_01_) was completely suppressed, even at the lowest GO concentration, thus clearly pointing to the formation of highly effective electronic interface interactions between both components. Likewise, the intensity of the *A*″_00_ shoulder at 231 nm is strongly reduced at the lowest GO concentration and progressively decreases further with increasing GO concentration, eventually resulting in its entire loss. The *S*_2_ and *S*_1_ states face a similar intensity decrease. Moreover, the superimposed vibronic peaks (*A*_00_, *A*_01_, *A*′_00,_ and *A*′_01_) undergo important changes as expressed by a systematic enhancement of the *A*_00_/*A*_01_ intensity ratio with increasing GO concentration. Reaching a value of 1, and also exhibiting small wavelength shifts of about 8 nm toward higher wavelengths, is a clear indication that NH_2_-X molecules adopt a more planar conformation when electronically interfaced with GO via π–π stacking and/or hydrogen bonding, a situation very similar to the case of conjugated polymers.^21,22^ A more detailed discussion on vibrational changes can be found in Supporting Information, Section S3. The established electronic interface interactions in NH_2_-X–GO not only affected the characteristic excited singlet states of NH_2_-X but also its fluorescence behavior. Figure 2c shows the fluorescence emission spectra of NH_2_-X–GO upon GO addition, acquired at an excitation wavelength of 480 nm, which falls in the range of the *S*_1_ state. As discussed above, the initial NH_2_-X spectrum exhibits a maximum emission at 528 nm, accompanied by a shoulder at 560 nm. NH_2_-X–GO reveals the same spectra as NH_2_-X itself, clearly underlining the nonfluorescent character of GO. However, their intensity is progressively quenched with increasing GO concentration. The same quenching behavior is found for emission spectra excited at 315 nm into the *S*_3_ state (Supporting Information, Figure S6), which also contributes to the fluorescence emission at 528 nm, as discussed above. Therefore, with increasing GO content, the established electronic interaction effectively suppresses the fluorescent *S*_1_–*S*_0_ channel and its contributing internal conversion pathways from the *S*_2_ and *S*_3_ molecular states. The quenching process of the NH_2_-X chromophore was further evaluated by the Stern–Volmer analysis. Figure 2d reveals a linear relationship between the ratio of the initial fluorescence intensity (*I*_0_) to the final fluorescence intensity (*I*_f_) versus GO concentration. From the slope, a Stern–Volmer constant of *K*_SV_ = 25.42 L/g was determined. With a measured fluorescence lifetime of the NH_2_-X chromophore of τ = 2.94 ns, a fluorescence rate constant *k*_q_ = 8.65 × 10^9^ g/L·s is obtained (more details on the analysis are provided in the Supporting Information, Section S5 and S6). This value falls in the characteristic range of static quenching processes,^32^ thus indicating the formation of physical aggregates of the NH_2_-X–GO system.

**Figure 2:**
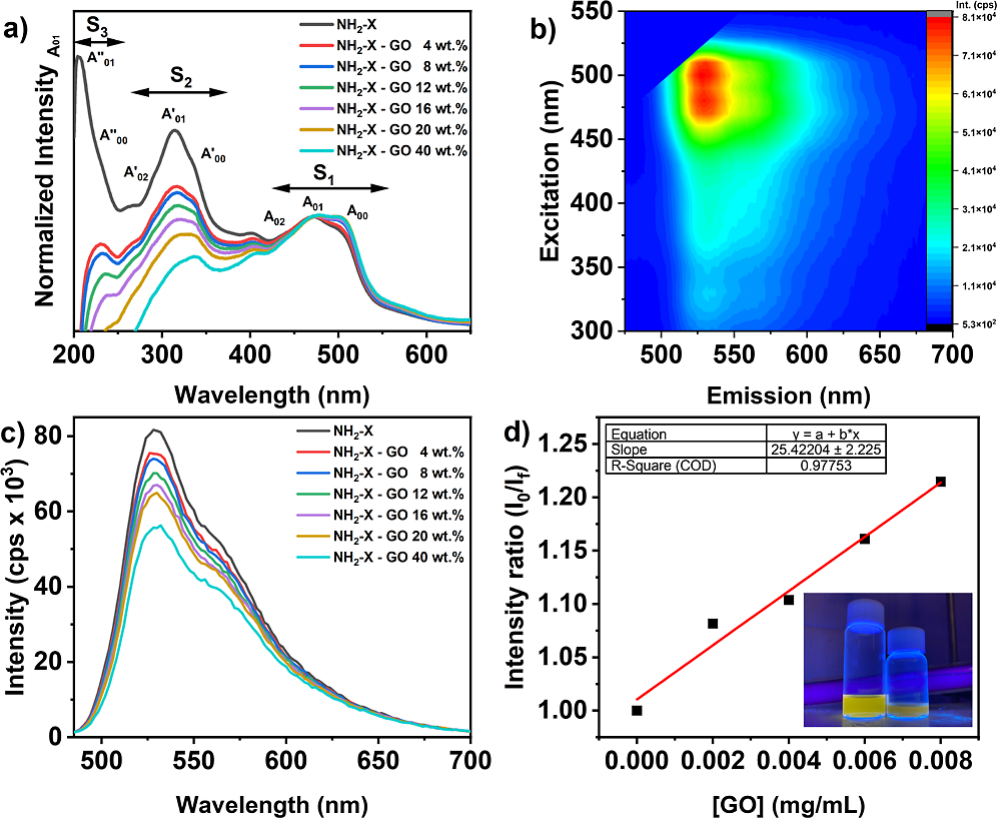
Photophysical characterization. (a) UV–vis spectra of NH_2_-X–GO at different GO concentrations. The hybrid spectra are GO-corrected and normalized to the S_1_ singlet maximum at 472 nm of the original NH_2_-X spectrum. (b) 2D fluorescence excitation–emission map of the NH_2_-X dye molecule. (c) Fluorescence emission spectra of NH_2_-X–GO at different GO concentrations, excited at 480 nm. d) Stern–Volmer analysis, whereby black squares indicate experimental data points and red line indicates the linear fitting. Fitting results, as well as a photograph of the fluorescent NH_2_-X solution (left) and partially quenched NH_2_-X–GO solution (right), are included as the inset.

Such a physical association probably involves the adsorption of NH_2_-X on GO, most likely via π–π stacking between the NH_2_-X dye molecule and planar sp^2^ graphene domains of GO sheets, inducing flattering of the xanthene core. This may be accompanied by hydrogen bonding or dipole interactions between the NH_2_- residue and oxygen functional groups (−OH, –COOH) of GO, restricting NH_2_- rotational modes and thus eventually leading to a more coplanar geometry with the xanthene ring structure. The planarization effect of the NH_2_-X molecules associated with GO is well expressed by the increased intensity of the *A*_00_/*A*_01_ ratio in the UV–vis spectra involving the related key vibrational modes. Moreover, it favors electronic coupling between the dye and the GO π-system. The suppression of excited singlet states, going along with slight shifts toward lower energy values, is consistent with electronic delocalization and the formation of a NH_2_-X–GO ground-state charge-transfer hybrid. This effectively transfers photoexcited electrons from the NH_2_-X to the GO conduction band (or π*-states), thus suppressing the molecular radiative decay. It is noteworthy that the observed concentration dependency of the spectroscopic changes reflects the increase of the interaction possibilities of NH_2_-X and GO in solution with enhanced GO concentration. Table 1 gathers the main spectroscopic observations, while Figure 3 shows the absorption and fluorescence mechanism of both the NH_2_-X molecule and the NH_2_-X–GO charge-transfer hybrid.

**Table 1:** Main Spectroscopic Observations: Positions of Electronic and Vibronic States of the NH_2_-X Dye Molecule and of GO, Along with Relevant Changes for NH_2_-X–GO Charge-Transfer Hybrids

clectronic transition NH_2_-X	position (nm)/(eV)	changes NH_2_-X–GO	Δ*E*_phonon_ (eV)	comments
S_1_				electronic transition (π-π*), responsible for *S*_1_–*S*_0_ fluorescence
*A*_00_	496/2.50	*A*_00_/*A*_01_ ratio enhancement^a^	∼ 0.13	vibrations involving ring breathing modes: (C–H)_in‑plane bending._, (N–H)_bending_, (C–N)_sp2_ _stretching_, (NH_2_)_bending_, (C–C)_aromatic stretching._ and combinations
*A*_01_	472/2.63			
*A*_02_	443/2.80		∼ 0.17	
–	403/3.08			electronic transition (+n–π*mix.)
S_2_				higher electronic transition (π–π*)
*A*′_00_	335/3.70	*A*′_00_/*A*′_01_ ratio enhancement^a^	∼ 0.24	vibrations involving ∼ double-frequency combinations
*A*′_01_	315/3.94			
*A*′_02_	293/4.23		∼ 0.29	
–	268/4.63			higher electronic transition (+n–π*)
S_3_				higher electronic transition (π–π*)
*A*″_00_	231/5.37	reduced	∼ 0.71	vibrations involving higher-frequency combinations
*A*″_01_	204/6.08	suppressed		
GO				
*n*–π*	310/4.00			broad tail (high density of trap states)
π–π*	230/5.39			

a *A*_00_, *A*_01,_ and *A*_02_ additionally undergo slight shifts toward higher wavelengths with increasing GO concentration (maximum of about 8 nm).

**Figure 3:**
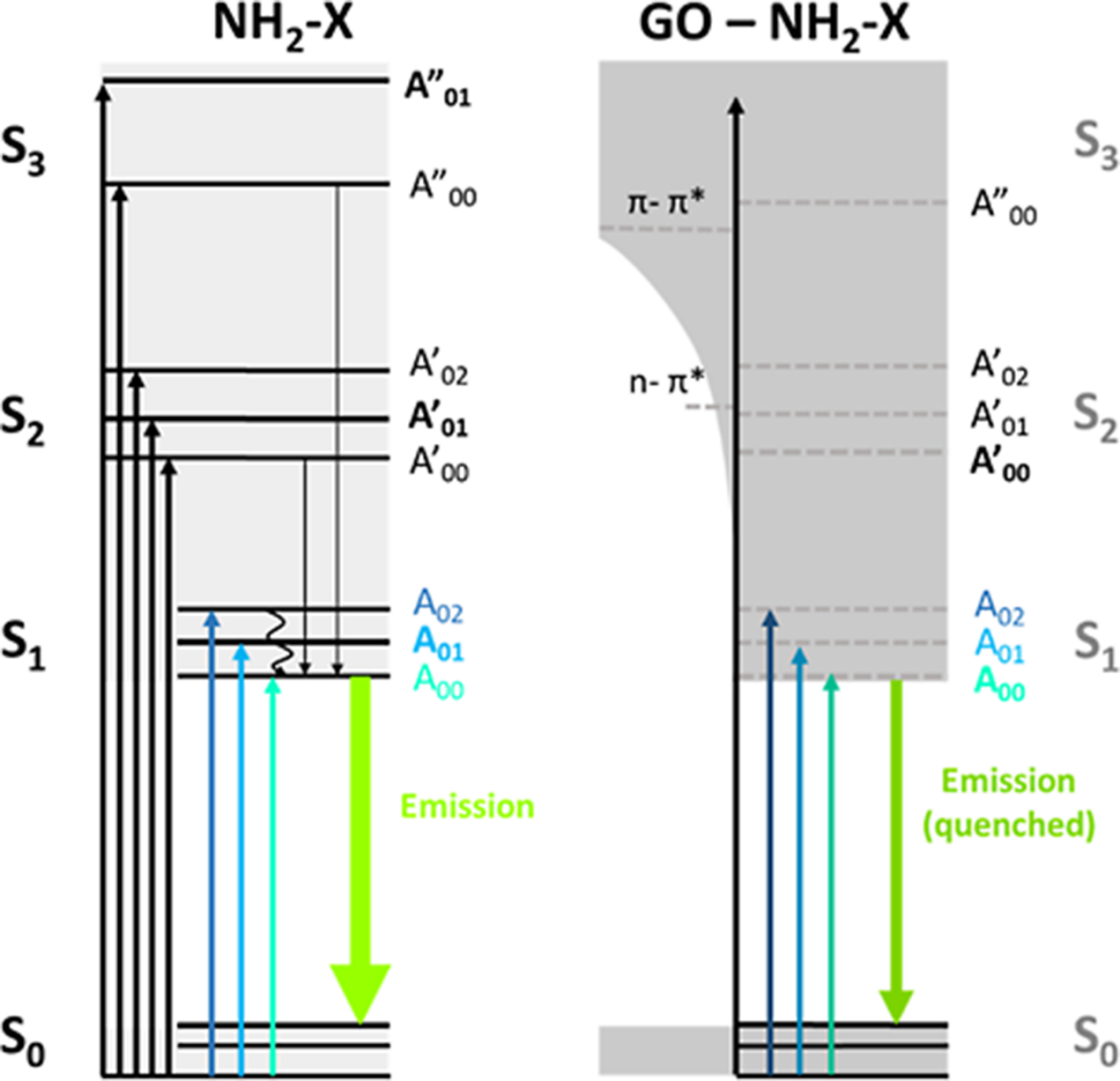
Absorption and fluorescence mechanism for the NH_2_-X dye molecule and NH_2_-X–GO ground state charge-transfer hybrid. Upward arrows denote transitions from singlet ground state S_0_ to higher excited singlet states *S*_n_, including associated vibration states *A*_0n_. Black lines from *S*_n_ to *S*_1_ denote nonradiative internal conversion processes, including nonradiative vibrational relaxations from *A*_0n_ to *A*_00_, contributing to the population of *S*_1_. The green downward arrow denotes the green fluorescence from *S*_1_ to *S*_0_. For the NH_2_-X–GO charge-transfer hybrid, the dark gray shaded area denotes the newly joint common electronic states. Weak dashed lines denote *S*_n_ of the original NH_2_-X molecular system. The upward arrow denotes transitions that are intensity-reduced or even suppressed in the final charge-transfer hybrid. π–π* and *n*–π* transitions of original noninteracting GO sheets are also indicated.

With its charge-transfer characteristics, the NH_2_-X–GO hybrid may offer enhanced performance as a sensitizing layer for photoanodes in PEC applications. To explore this opportunity, dispersions of NH_2_-X and NH_2_-X GO were employed in a spray coating process for subsequent casting onto FTO-supported TiO_2_ film substrates, resulting in NH_2_-X- and NH_2_-X GO-modified TiO_2_ photoanodes. Figure 4a displays the Raman spectra of the original TiO_2_ and modified TiO_2_ photoanodes, labeled TiO_2_/NH_2_-X and TiO_2_/NH_2_-X–GO. The spectrum of the TiO_2_ photoanode reveals the typical Raman signals at around 140 cm^–1^, 390 cm^–1^, 515 cm^–1^, and 640 cm^–1^ corresponding to the *E*_1__g_, *B*_1g_, *A*_1g_ + *B*_1g_, and *E*_3__g_ vibrational modes.^33^ These strong modes also dominate the spectra of the modified photoanodes. The TiO_2_/NH_2_-X shows superimposed broad Raman bands at about 1040 cm^–1^ and 1300 cm^–1^ with weaker intensity, highlighted in the inset of Figure 4a, which details the spectral features for Raman shifts beyond 600 cm^–1^. Their frequencies coincide with vibrational modes that are sensitive to the planarity of the NH_2_-X molecule, as identified in the UV–vis spectra. It is likely that the amino groups of NH_2_–X interact with acidic surface sites of TiO_2_ through hydrogen-bonding or coordination-type interactions, as observed for other nitrogen-containing dyes.^7^ As discussed above, the band at 1040 cm^–1^ and 1300 cm^–1^ can be assigned to the combinational vibrations of the xanthene ring breathing mode + (C–H)_in‑plane bending_ modes as well as combinations of (N–H)_bending_, (C–N)_stretching_, (NH_2_)_bending,_ and (C–C)_sp2 stretching_ modes, respectively. In the case of the TiO_2_/NH_2_-X GO photoanode, a decreased intensity of these two Raman bands is encountered, in agreement with the respective intensity changes observed in the UV–vis spectra. Furthermore, the presence of GO in this photoanode is clearly confirmed by the broad G-band at about 1590 cm^–1^ and the D-band at 1350 cm^–1^, which appears as a shoulder, being both distinct features of GO.^34^ Morphological inspection of these photoanodes was performed by acquiring FE-SEM images. Figure 4b reveals a granular surface morphology for the TiO_2_ reference photoanode, characteristic of the employed TiO_2_ paste. The TiO_2_/NH_2_-X photoanode does not disclose relevant changes (Figure 4c), indicating that the adsorbed NH_2_-X layer smoothly adopts to the underlying TiO_2_ film morphology, thus agreeing with observations on other types of dye-sensitized systems.^7^ On the contrary, the TiO_2_/NH_2_-X–GO photoanode (Figure 4d) reveals the presence of isolated micrometer-sized surface coverages. These visibly exhibit an internal morphology, unique to the presence of GO sheets most likely embedded in NH_2_-X. This indicates, once more, the close interface interactions established between both components in the charge-transfer hybrids. A possible intercalation between GO sheets may prevent self-aggregation and further contribute to favorable charge-transfer processes across the interfaces of the isolated hybrid spots, either being in direct contact with the TiO_2_ surface or located on NH_2_-X-coated areas originating from unbound NH_2_-X in the hybrid dispersion. More images on the random distribution of the isolated spots can be found in Supporting Information, Figure S8.

**Figure 4:**
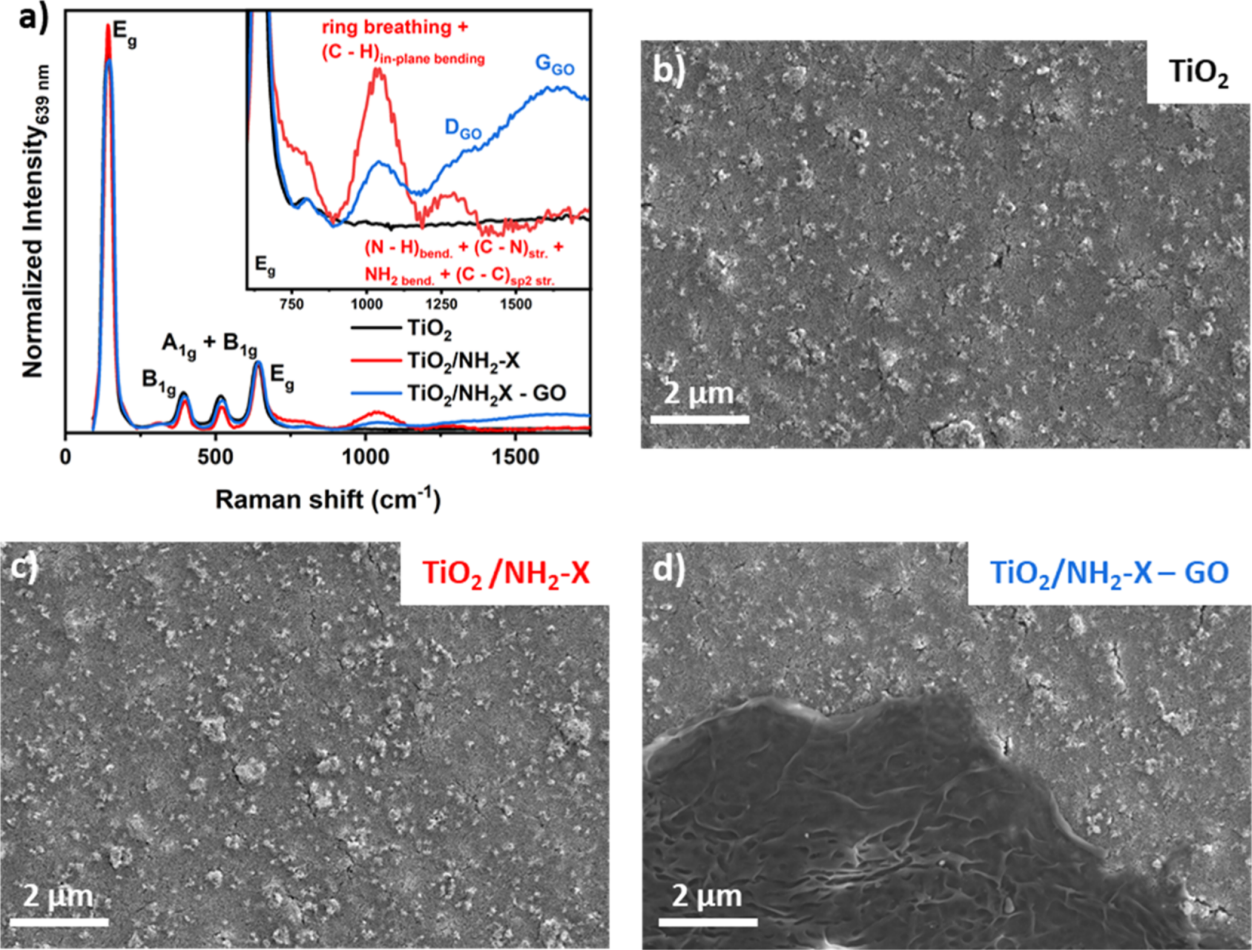
Characterization of the employed photoanode films. (a) Raman spectra of TiO_2_ (black), TiO_2_/NH_2_-X (red), and TiO_2_/NH_2_-X–GO (blue) acquired at λ_exc_ = 532 nm. FE-SEM images of (b) TiO_2_, (c) TiO_2_/NH_2_-X, and (d) TiO_2_/NH_2_-X–GO. The GO loading rate corresponds to 0.4 wt %.

Next, a systematic PEC characterization of the photoanodes was carried out (Figure 5). Results are presented for the optimized electrolyte composition and illumination parameters. The hybrid photoanode selected for these studies corresponds to a GO loading of 0.4 wt %. The preconditioning protocol to establish most favorable conditions can be found at Supporting Information, Sections S8–S10, as well as PEC measurements to isolate the role of GO in TiO_2_ photoanodes (Supporting Information, Section S11).

**Figure 5:**
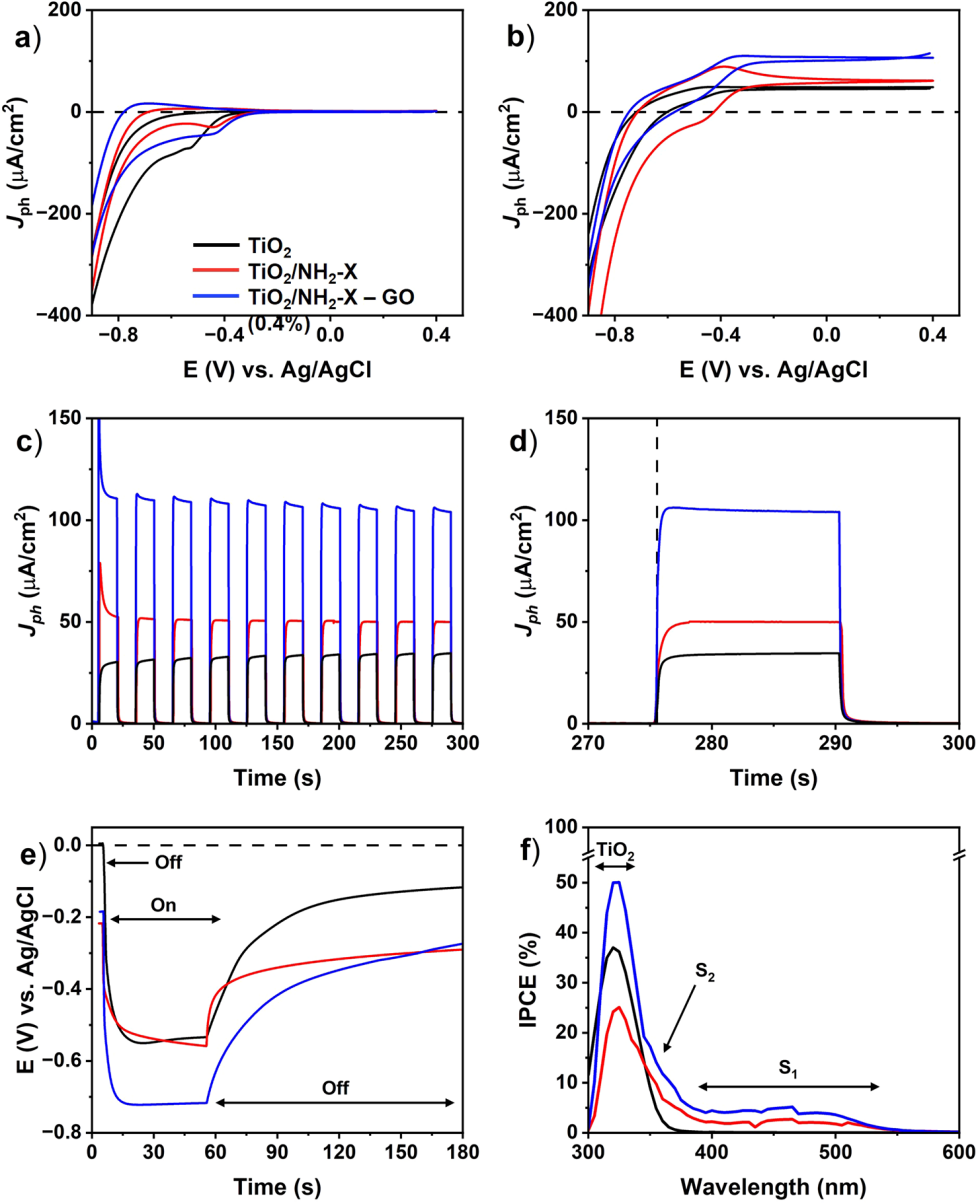
PEC characterization of the employed photoanodes. Cyclic voltammograms (a) in the dark and (b) illumination conditions. (c) Potentiostatic on–off pulse measurements at 0 V vs Ag/AgCl and (d) zoom-in of the last pulse. (e) Photopotential measurements and (f) IPCE spectra recorded at 0 V (vs Ag/AgCl). Illumination conditions: 25 mW/cm^2^.

Figure 5a,b depicts the results of cyclic voltammetry (CV) under dark and illumination conditions, respectively, for the bare and sensitized TiO_2_ photoanodes with NH_2_-X and NH_2_-X–GO. CV curves under dark conditions reveal the accumulation of charges for potentials below −0.3 V for TiO_2_, shifting to slightly less negative values for the sensitized photoanodes. A hump appearing at −0.5 V for TiO_2_, typically assigned to monoenergetic trap states,^35^ changes its position by approximately 50 mV to −0.45 V for both of the sensitized photoanodes. The lack of a corresponding feature discharging the system suggests that corresponding charges remain trapped in these states.^35^ Slight variations in shape of the charge accumulation region indicate changes in the photoanode surface, likely due to the modification of the surface chemistry induced by the presence of NH_2_-X and NH_2_-X–GO hybrids. Cyclic voltammograms under illumination (Figure 5b) depict important photocurrent generation at potentials above −0.5 V. In analogy to the nonilluminated conditions, this potential shifts to less negative values for the sensitized photoanodes, resulting in enhanced photocurrent generation, compared to the bare TiO_2_ photoanode. At a potential of 0 V, the photocurrents are stabilized, whereby the NH_2_-X–GO sensitized photoanode exhibits photocurrents more than twice as high compared with the other two photoanodes. This provides a favorable situation for their exploitation under PEC water splitting conditions. The observed enhancement of the photocurrent is most likely related to efficient charge separation and extraction of photogenerated charges, promoted by the presence of the xanthene dye and beneficial conductive pathways provided by GO for the hybrid material.

Transient photocurrent measurements with repeated on–off pulses of 15 s recorded over a time of 5 min are shown in Figure 5c. The photocurrent of the TiO_2_ photoanode is characterized by a relatively slow rise in the photocurrent until reaching a plateau, which stabilizes during the following pulses at slightly higher photocurrent values. This situation differs for the modified TiO_2_/NH_2_-X and TiO_2_/NH_2_-X–GO photoanodes. Both reveal a strong overshooting photocurrent peak being a characteristic feature of dye-sensitized photoanodes related to electron recombination processes with trap states or photooxidation intermediates.^7^ The photocurrent is then stabilized until reaching a plateau level that is significantly higher than that of bare TiO_2_, offering values increased by a factor of about 3.5 for the TiO_2_/NH_2_-X–GO photoanode. Importantly, from the second pulse on, the photocurrents already show saturation while the shape characteristic resembles that of the nonmodified TiO_2_ photoanode, thus revealing improved charge-transfer effects due to the additional xanthene coating layer. Moreover, a magnified zoom of the last pulse (Figure 5d) shows a faster response time toward photocurrent saturation for the TiO_2_/NH_2_-X–GO photoanode (0.5 s) compared to the TiO_2_/NH_2_-X (4 s) and TiO_2_ (2.5 s). This observation thus points to relevant changes in the kinetics of photoinduced charge carrier processes, being most advantageous when sensitizing TiO_2_ photoanodes with the NH_2_-X–GO charge-transfer hybrids.

More insight into processes related to the accumulation and separation of photoinduced charge carriers is obtained by time-dependent measurements of the photopotential (*E*_ph_), denoted as the difference between the open circuit potentials acquired under dark (OCP_dark_) and illuminated (OCP_light_) conditions. Figure 5e displays the OCP behavior under on–off conditions for each photoanode. At *t* = 0 s, an OCP_dark_ value for TiO_2_ of 0 V is encountered, whereas TiO_2_/NH_2_-X and TiO_2_/NH_2_-X–GO photoanodes reveal values shifted to −0.21 and −0.19 V, respectively. At *t* = 55 s, the OCP_light_ values are −0.53 V for TiO_2_ and −0.56 V for NH_2_-X/TiO_2_, while the TiO_2_/NH_2_-X–GO exhibits an enhanced value of −0.71 V. This results in *E*_ph_ values of −0.53 V, −0.35 V, and −0.52 V for the TiO_2_, TiO_2_/NH_2_-X, and TiO_2_/NH_2_-X–GO photoanodes, respectively (see Table 2 for a summary of recorded PEC parameters). The establishment of a photopotential indicates band bending at the semiconductor/electrolyte interface, proving that photogenerated charge carriers separate and, thus, contribute to *E*_ph_. The negative signs of the photovoltages imply that the employed photoanodes overall behave as *n*-type semiconductors. This is related to the presence of the TiO_2_ layer itself, where oxygen vacancies generated within the crystalline lattice donate extra electrons to the bottom edge of the conduction band.^36^ Interestingly, the absolute *E*_ph_ value for the TiO_2_/NH_2_-X photoanode exhibits a lower value, indicating a less effective separation of photoinduced charge carriers, most likely due to recombination losses at the excited dye and poor charge-transfer kinetics within the TiO_2_/NH_2_-X solid–solid (*S*–*S*) interface, as confirmed by a nonstabilized OCP_light_ value at *t* = 55 s. However, the TiO_2_/NH_2_-X–GO photoanode recovers the value of bare TiO_2_, potentially caused by the enhanced charge-transfer kinetics offered by the NH_2_-X–GO charge-transfer hybrid. Upon switching off the light at *t* > 55 s, the OCP_dark_ behavior reflecting charge recombination kinetics provides further evidence to this assumption. In particular, the TiO_2_/NH_2_-X–GO photoanode exhibits a faster OCP recovery toward the initial OCP_dark_, thereby offering improved charge mobility of photoinduced charge carriers in the dark compared to TiO_2_/NH_2_-X. Furthermore, the TiO_2_/NH_2_-X displays a quite different relaxation characteristics with respect to the TiO_2_/NH_2_-X–GO photoanode, which resembles the one of bare TiO_2_. This suggests that the enhanced charge mobility within the NH_2_-X–GO hybrid assists in overcoming the solid–solid interface resistance created by the NH_2_-X coverage layer on TiO_2_.

**Table 2:** Relevant PEC Parameters^a^

photoanode	*J*_ph_ (μA/cm^2^)	*t*_saturation_ (s)	OCP_dark_ (V)	OCP_light_ (V)	*E*_ph_ (V)
TiO_2_	33	2.5	0	–0.53	–0.53
TiO_2_/NH_2_-X	51	4	–0.21	–0.56	–0.35
TiO_2_/NH_2_-X–GO	105	0.5	–0.19	–0.71	–0.52

a Photocurrent (*J*_ph_) and saturation time (*t*_saturation_) refer to the last pulse.

In addition to the improved charge-transfer kinetics, sensitizing effects that involve enhanced light absorption ability also account for substantial enhancement of the generated photocurrent. Therefore, the sensitization process is analyzed through the incident photon-to-current efficiency (IPCE) spectra shown in Figure 5f. The IPCE spectrum of bare TiO_2_ reveals its characteristic absorption in the UV range between 300 and 370 nm, exhibiting its maximum at 320 nm, in agreement with the literature.^37^ For the TiO_2_/NH_2_-X and TiO_2_/NH_2_-X–GO photoanodes, the performance range is extended toward the visible range, showing a light absorption contribution originating from the S_1_ transition of the NH_2_-X molecules, supporting a clear sensitization effect. Curiously, at first sight, the NH_2_-X–GO hybrid reveals an even increased light harvesting performance despite its lower *S*_1_ absorption (see the discussion above). Apparently, the favorable charge transport kinetics of the hybrid comes into play, compensating for its reduced absorption characteristics. In addition, a significantly enhanced absorption performance is observed at around 345 nm for both sensitized photoanodes. This improvement is ascribed to the strong *S*_2_ light absorption of NH_2_-X, thus fully underlining the sensitization effect. However, since the IPCE value at this wavelength is higher for the TiO_2_/NH_2_-X–GO photoanode, despite its suppressed absorption behavior, the advantageous charge-transfer kinetics of the hybrid must account for the enhanced IPCE at these wavelengths. These results thus reveal a unique interplay between sensitization and charge-transfer kinetics offered by the NH_2_-X–GO hybrid layer, contributing to the overall enhancement of the photocurrent for the modified photoanodes.

Further analysis of the interface processes is provided by electrochemical impedance spectroscopy (EIS). Figure 6 shows Bode and Nyquist plots acquired for each photoanode under dark and illumination conditions at their respective OCP_dark_ and OCP_light_ values. The Bode plot obtained under dark conditions (Figure 6a) shows a broad phase angle band for all employed photoanodes reaching maximum values at low frequencies, which are typical for processes occurring at the solid–liquid electrode/electrolyte interface.^37–39^ Of all three photoanodes, TiO_2_ exhibits the highest phase angle value of almost 90°. While this is reached in a rather slow plateauing manner from 3 Hz on, TiO_2_/NH_2_-X and TiO_2_/NH_2_-X–GO expose well-defined maxima at about 2 Hz. Overall, with phase angles near 90°, the general characteristics of the Bode plots reveal charge processes described by an almost ideal capacitor behavior at the solid–liquid interface. The Nyquist diagrams under dark conditions (Figure 6b) show impedance behaviors characterized by semicircles with large arc diameters, indicating an overall high charge separation resistance for each of the photoanodes. Under illumination conditions, the situation changes significantly. The respective Bode plots (Figure 6c) depict a smaller phase angle band with more defined maxima at low frequencies. Moreover, a systematic decrease of the maximum phase angle value is observed, with TiO_2_ showing values below 80°, TiO_2_/NH_2_-X around 60°, and TiO_2_/NH_2_-X–GO near 50°. The Bode plot maximum of TiO_2_ is shifted toward a lower frequency of 0.3 Hz upon illumination, observing the same effect for TiO_2_/NH_2_-X–GO, whereas TiO_2_/NH_2_-X acquires an intermedium position at about 0.5 Hz. Such behavior is highly consistent with former observations of enhanced charge-transfer processes for the TiO_2_/NH_2_-X–GO photoanode, whereby the charge-transfer hybrid contributes to overcome the lower efficiency of the TiO_2_/NH_2_-X photoanode and recovers the original maximum frequency value of TiO_2_. Moreover, a shoulder of the Bode plot at frequencies close to 10^3^ Hz suggests charge separation processes taking place within the solid–solid interface of the photoelectroactive layer. The particular decrease in phase angle of the TiO_2_/NH_2_-X–GO photoanode further underlines the efficient charge separation and transport of photoinduced carriers ascribed to the charge-transfer hybrid. The Nyquist plots under illumination (Figure 6d) reveal systematic lowering of the large semicircle arc for each photoanode, eventually leading to reduced charge mobility resistance. Importantly, a small semicircle appears at higher frequencies between 100 and 200 Ω for all photoanodes (see the inset in Figure 6d). In particular, the TiO_2_/NH_2_-X–GO photoanode exhibits the smallest semicircle within the series, clearly denoting improved charge separation of the photoinduced charge carriers. The experimental EIS data are well fitted (solid lines in both Bode and Nyquist plots) by employing the equivalent circuit shown in Figure 6e, best defining electrical transport phenomena in layered semiconducting electrodes.^37,39^ It is composed by a series resistance (*R*_S_) of the system along with two RC circuits in series, representing, respectively, charge transport processes at the solid–solid interface, described by resistance *R*_SS_ and constant phase element CPE_SS_. Electrode/electrolyte interfaces are expressed by *R*_SL_ and CPE_SL_. Table 3 summarizes the fitting values for each element involving each photoanode under dark and light illumination conditions.

**Figure 6:**
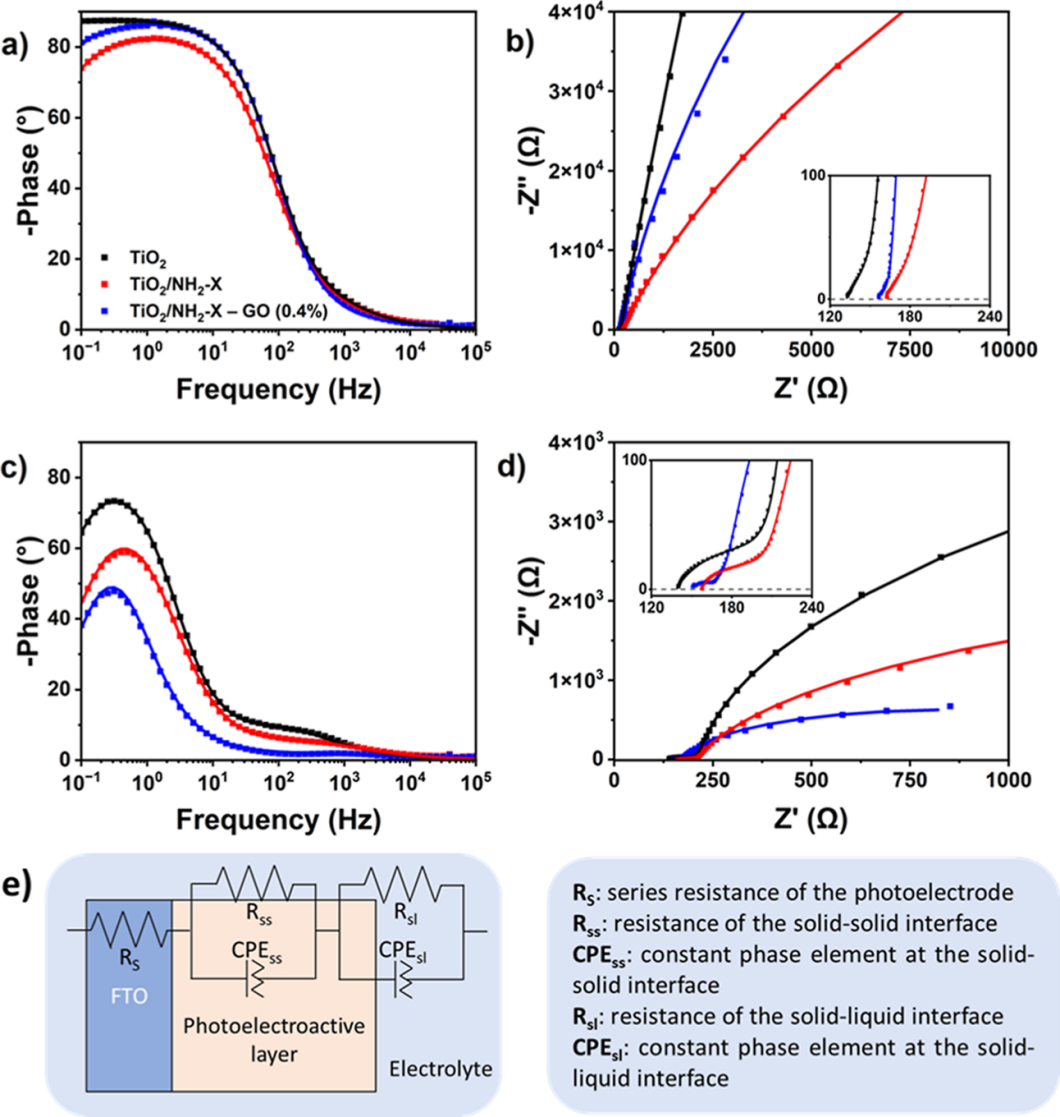
EIS measurements. (a) Bode and (b) Nyquist plots under dark; (c) Bode and (d) Nyquist plots under illumination. Lines represent fitted curves. (e) Employed equivalent circuit and description of its elements.

**Table 3:** Fitting Parameters of the Employed Equivalent Circuit from EIS Measurements for Each Photoanode under Both Dark and Illumination Conditions^a^

photoanode	OCP (V)	υ_Bode_ (Hz)	*R*_S_(Ω)	*R*_SL_(Ω)	*R*_SS_(Ω)	*C*_SL_(μF)^b^	*C*_SS_(μF)^b^
TiO_2_ (dark)	0	0.6	132	5.3 × 10^6^	32	15	268
TiO_2_ (light)	–0.53	0.3	138	5 × 10^4^	55	393	199
TiO_2_/NH_2_-X (dark)	–0.21	1.2	160	1.8 × 10^7^	25	12	93
TiO_2_/NH_2_-X (light)	–0.57	0.5	156	1.4 × 10^4^	77	298	160
TiO_2_/NH_2_-X–GO (dark)	–0.19	1.2	154	1 × 10^6^	12	12	210
TiO_2_/NH_2_-X–GO (light)	–0.71	0.3	145	1.3 × 10^3^	45	1250	395

a Values of OCP as well as frequency of the phase angle maximum are also indicated.

b Capacitance (*C*) values were obtained from fitted *Z*_CPE_ values *via Z*_CPE_ = 1/(*i*ω*C* ^α^), ω being the angular frequency, α being an empirical constant between 0 and 1, and *i* being the imaginary number.

The quantitative fitting values confirm the former qualitative discussion of the involved charge separation and transport phenomena. High *R*_SL_ values under dark conditions clearly reveal the absence of charge separation processes. A significant decrease of *R*_SL_ by 2 or 3 orders of magnitude is observed under illumination conditions, reflecting an effective separation of photoinduced charges at the solid–liquid interface. In particular, the lowest *R*_SL_ value is obtained for the TiO_2_/NH_2_-X–GO photoanode, owing to the favorable charge-transfer properties of the hybrids. Equally, TiO_2_/NH_2_-X–GO displays the lowest *R*_SS_ value, suggesting enhanced charge separation and transport at the solid–solid photoanode interface, overcoming the highest *R*_SS_ value of TiO_2_/NH_2_-X due to the favorable charge transport within the hybrid material. Moreover, under illumination conditions, the *C*_SL_ value of the TiO_2_/NH_2_-X–GO photoanode experiences an enhancement by a factor of almost 4 compared to bare TiO_2_, thus reflecting an increased amount of separated photoinduced charges that accumulate on the solid–liquid interface. On the contrary, the lowest *C*_SL_ value observed for the TiO_2_/NH_2_-X photoanode reveals limited efficiency to separate photoinduced charges, most likely leading to recombination processes that hinder straightforward charge transport. This finding supports the favorable charge-transfer action obtained by the NH_2_-X–GO hybrids. Finally, similar trends are found regarding *C*_SS_ values, indicative of building up space charge regions inside the solid–solid interface. The highest value is obtained for the TiO_2_/NH_2_-X–GO photoanode, being fully consistent with all charge separation and transport phenomena described and the overall advantageous charge-transfer ability of the developed NH_2_-X–GO hybrid.

## Conclusions

Enamino-xanthene dye molecules (NH_2_-X) and graphene oxide (GO) were successfully interfaced by employing a simple liquid-phase mixing process. Photophysical analyses reveal that upon the physical association, NH_2_-X molecules adopt a planar conformation on GO, which enables strong electronic interfacial interactions. These result in altered electronic transitions and fluorescence quenching, indicative of the formation of a ground-state NH_2_-X–GO charge-transfer hybrid material. When applied as a sensitizing layer onto TiO_2_ photoanodes, these hybrids improve light absorption in the visible range and facilitate rapid and efficient charge separation and transport. PEC characterization reveals a 3.5 increase in photocurrent, rapid saturation kinetics, and enhanced photopotential generation, i.e., efficient separation and carrier transport of photoinduced charges at both the solid–solid and the solid–liquid interfaces of the photoanode modified with the NH_2_-X–GO hybrid material. Since xanthenes constitute a family of environmental-benign dyes, our results underscore the potential of xanthene–GO hybrids as sustainable and effective sensitizers for advanced PEC applications.

## Supplementary Material



## Notes

Synthesis and characterization of NH_2_-X and GO; photoluminescence emission spectra; fluorescence lifetime and quenching mechanism of NH_2_-X–GO; FE-SEM of TiO_2_/NH_2_-X–GO photoanodes; and additional information on irradiation conditions and stability (PDF)
