# Supplementary material for: Graphene Oxide–Enamino-Xanthene Charge-Transfer Hybrids as High-Performance Sensitizing Interfaces for TiO2 Photoanodes
Source: ACS Appl Mater Interfaces. 2025 Dec 31;18(1):3172–82. doi: 10.1021/acsami.5c20186 (PMC12781099; doi:10.1021/acsami.5c20186)
Supplement: Supplementary file 1 [file am5c20186_si_001.pdf]

Supporting Information

Graphene Oxide – Enamino Xanthene Charge Transfer Hybrids as High-Performance Sensitizing Interfaces for TiO_2 Photoanodes

Carlos Martínez-Barón,[1] Juan Manuel Garrido-Zoido,[2] Miguel Á. Álvarez Sanchez,[1] Pedro Cintas,[2] Juan C. Palacios,[2] Alejandro Ansón-Casaos,[1] María Victoria Gil,[2] Ana M. Benito,[1] Wolfgang K. Maser[1*]

[1]Instituto de Carboquímica, ICB-CSIC, 50018 Zaragoza, Spain

[2]Departamento de Química Orgánica e Inorgánica, Facultad de Ciencias, and Instituto del Agua, Cambio Climático y Sostenibilidad (IACYS), Universidad de Extremadura, 06006 Badajoz, Spain

*Corresponding author: wmaser@icb.csic.es

Table of Contents

Section S1.Synthesis and characterization of enamino-xanthenes................................S3

Section S2. Characterization of graphene oxide...S5

Section S3. UV-Vis spectra of NH_2-X and NH_2-X – GO S7

Section S4. UV-Vis spectra of GO...S9

Section S5. Photoluminescence emission spectra of NH_2-X – GO at 315 nm.............S10

Section S6. Fluorescence lifetime of NH_2-X and quenching mechanism.....................S11

Section S7. FE-SEM of TiO_2/NH_2-X – GO photoanodes S14

Section S8. Preconditioning of the hybrid photoanodes S15

Section S9. Effect of illumination conditions...S18

Section S10. Stabilization of dye sensitized photoanodes...S19

Section S11. Isolated role of GO in TiO_2 photoanodes...S21

References...S22

Section S1. Characterization of enamino xanthene

The synthetic procedure and subsequent characterization of the employed enamino xanthene has been previously reported.[1] Here, we include the specific ^1H NMR, HRMS results for *2-(Aminomethylene)-6,8-dihydroxy-1H-xanthene-1,3(2H)-dione (NH_2-X):*

^1H NMR (DMSO-*d6*, 500 MHz) δ (ppm) 10.55 (d, J = 17.0 Hz, 2H), 10.52 (d, J = 15.6 Hz, 2H), 9.32 (s, 2H), 8.25 – 8.10 (m, 4H), 6.20 (s, 2H), 6.14 (s, 2H), 5.53 (s, 1H), 5.50 (s, 1H).

HRMS (Electrospray ionization, positive mode), $C_{14}H_9NO_5$ $[M+H]^+$ *m/z* calcd. 272.0554, found 272.0545 (see Figures S1 – S2).

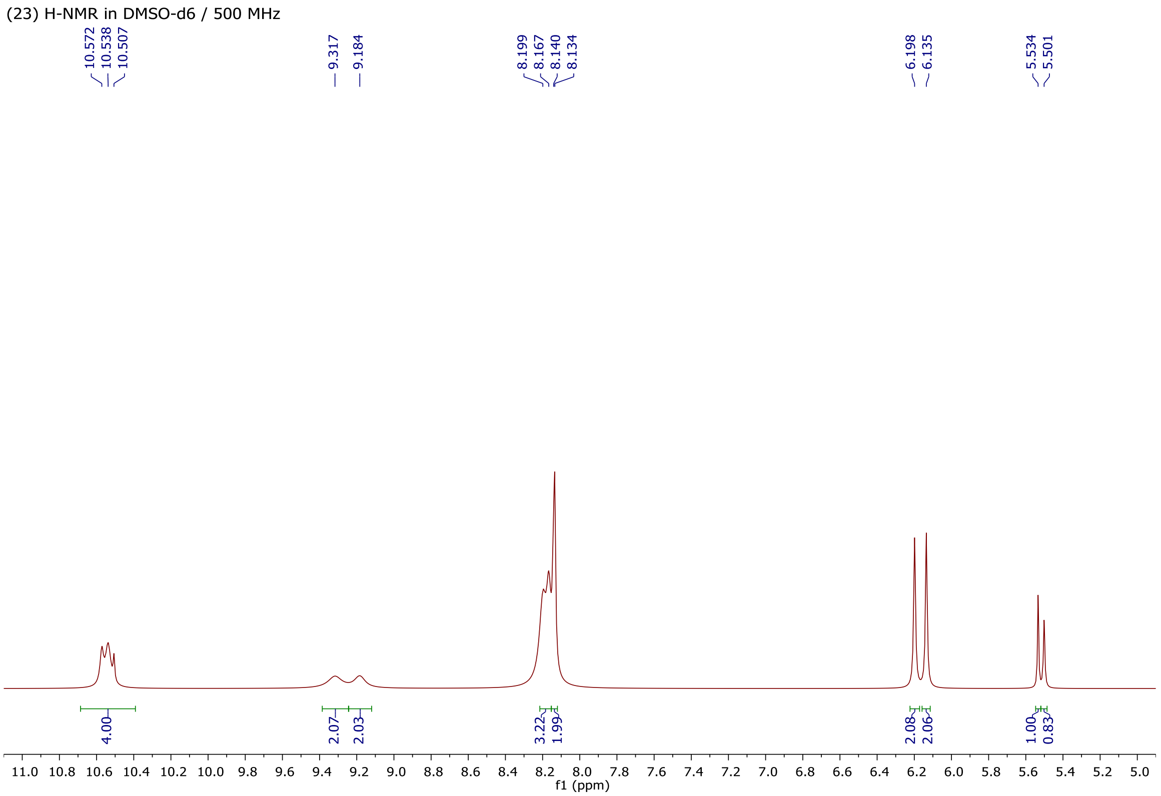

Figure S1. ^1H NMR spectrum of the NH_2-X compound.

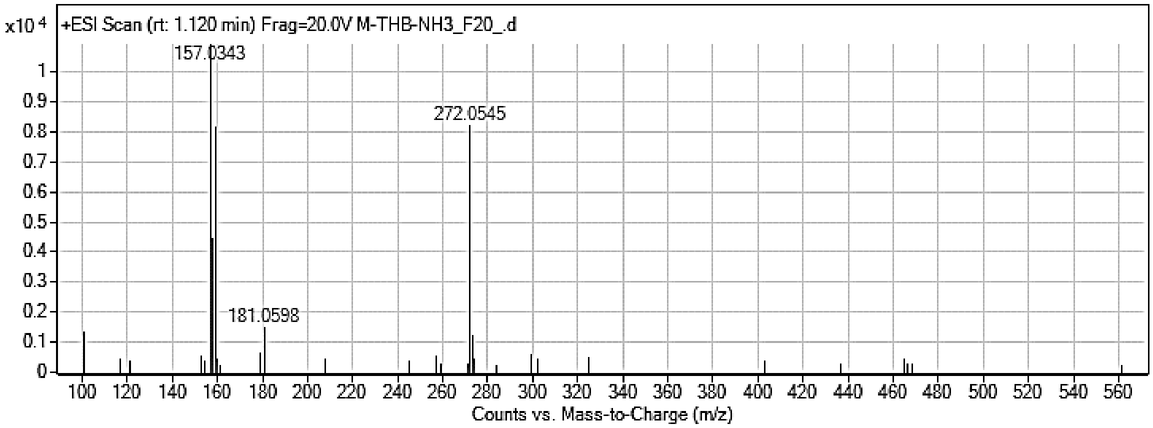

Figure S2. HRMS (ESI positive mode) of the NH_2-X compound. $[M+H]^+$ *m/z*: 272.0554 (calcd); found: 272.0545.

Section S2. Characterization of graphene oxide

The employed graphene oxide was synthesized following a modified Hummers protocol.[2,3] Figure S3 shows the physicochemical characterization of such nanomaterial. The XRD data (Figure S3a) reveal a prominent peak at about 11º which corresponds to the (001) interplanar distance of restacked individual GO sheets containing physiosorbed and chemisorbed water molecules and organized in a turbostratic manner.[4] Furthermore, a very weak peak at 21º is also detected and ascribed to the (002) interplanar plane graphite, indicating the presence of some very low amounts of remaining non-oxidized and non-exfoliated graphite precursor flakes. [5,6] These findings clearly indicate the presence of highly oxidized and well exfoliated GO sheets. This fact was corroborated according to the Raman spectra (Figure S3b). The presence of G–band (1612 cm^{-1}) and 2D–band (2750 cm^{-1}) are characteristic of the C_{sp2}–C_{sp2} bonds in graphene.[7,8] The 2D–band located at 2750 cm^{-1} consist on a second-order Raman feature of the D–band arising from a double-resonance process.[9] Moreover, the presence of the D–band at 1350 cm^{-1} reveals the existence of structural imperfections induced by the attachment of oxygen functional groups (OFGs) at the carbon basal plane.[8] Indeed, the high D / G ratio and the width of the 2D–band are also indicative of the high oxidation degree. Further characterization of the OFGs is shown at the FTIR spectrum in Figure S3c, whose most relevant peaks are assigned in agreement with GO literature.[10,11] The C=O stretching modes (1808 and 1726 cm^{-1}), water scissor mode (1618 cm^{-1}), O–H in-plane bending modes (1403 and 1378 cm^{-1}), C–O stretching modes (1226, 1052 and 1002 cm^{-1}), C–O–C bending mode (835 cm^{-1}) and water vibration mode (586 cm^{-1}). Thermogravimetric analysis under N_2 atmosphere is shown in Figure S3d. Two important mass losses are detected in the temperature range from 30 to 130 ºC and between 130 and 260 ºC. The first mass loss is

normally assigned to the desorption of physiosorbed water intercalated between the GO sheets, whereas the second mass loss is typically attributed to the decomposition of the OFGs into CO, CO_2 and steam.[4,12] Indeed, GO mass losses of 5 % and 35 % are encountered for the two regions, respectively.

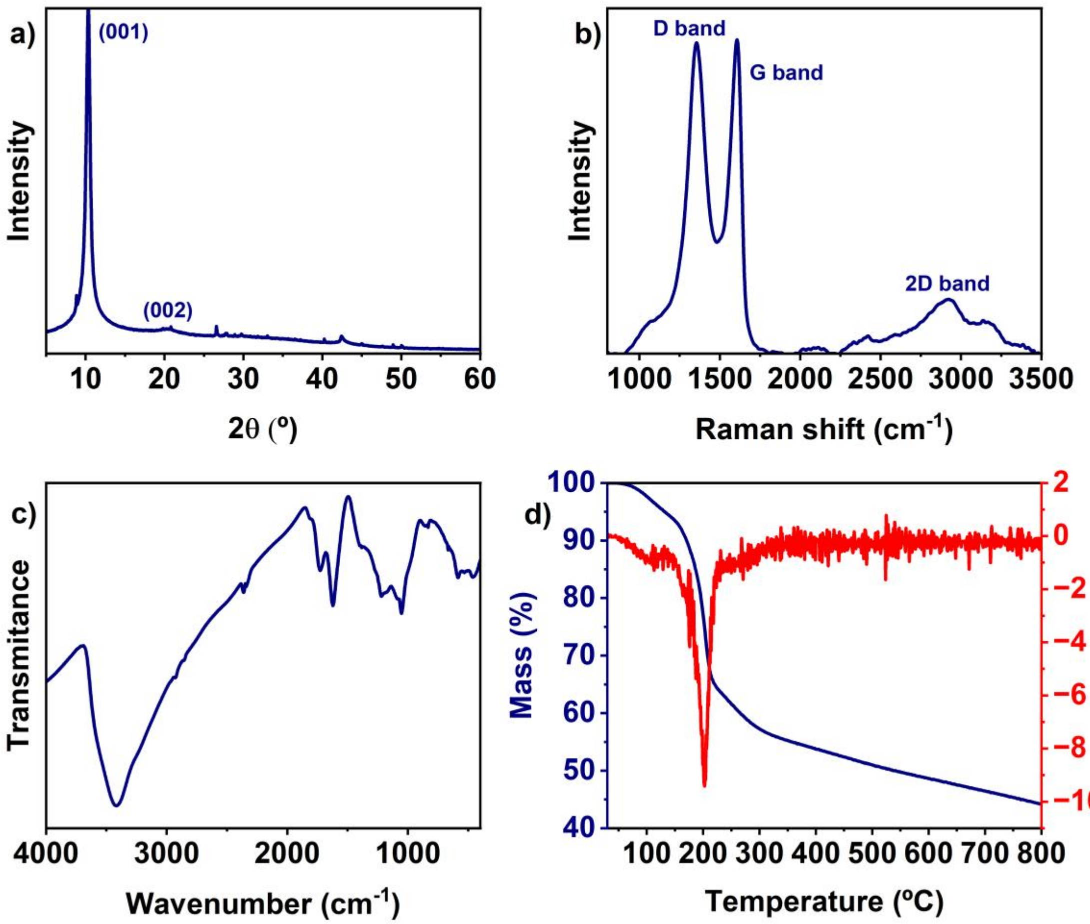

Figure S3. GO characterization. (a) XRD pattern, (b) Raman spectra at 514 nm, (c) FTIR spectrum and (d) TGA curve under N_2 atmosphere.

Section S3. UV-Vis spectra of NH_2-X and NH_2-X – GO

NH_2-X spectra and GO were discussed in the main manuscript and Supporting Information, respectively. For each GO concentration, the overall absorption intensity of the GO spectra was below the one of the corresponding NH_2-X transitions (Figure S4a). While the intensity of the GO spectra scales with concentration, it only provides a proportional additive offset to the featureless region of the NH_2-X – GO spectra beyond 600 nm. No further feature of the GO spectra itself was observed in the NH_2-X – GO spectra, belonging to the transitions of the NH_2-X molecule itself. This suggests a strong interaction between NH_2-X and GO when brought into contact. The spectroscopic consequences were described in detail in the main manuscript, at hand of the GO corrected spectra, which corresponds to the removal of the non-interacting GO contribution, achieved by subtraction of the GO spectra at a given concentration. For a meaningful discussion, the resulting spectra were further normalized to the maximum intensity of the S_1 band of the original NH_2-X spectrum, corresponding to its A_{01} vibrational mode at 472 nm.

In particular, the S_1 band of the original NH_2-X spectra is centered at around 480 nm revealing fundamental vibronic A_{00}, A_{01} and A_{02} quanta at 496 nm (2.50 eV), 472 nm (2.63 eV) and 443 nm (2.80 eV) with A_{01} exhibiting the highest intensity (Figure S4b), denoting a typical situation for conjugated systems. The phonon energies correspond to values of 0.13 eV (1049 cm^{-1}) and 1371 cm^{-1}. Spectra were normalized to the A_{01} intensity, serving as reference point for analyzing the effects of GO in the NH_2-X – GO hybrid spectra. Importantly, upon addition of GO, the intensity ratio of A_{00} / A_{01} increased, reaching a value of 1. This enhancement strongly reminds on the interaction of GO with conjugated polymers,[13,14] indicating a conformational change of the NH_2-X molecule to a more planar

structure. The observed vibrations with frequencies at 1049 cm^{-1} and 1371 cm^{-1}, associated to modes highly sensitive to planarity (i.e., ring breathing, $(C–H)_{in\text{-}plane\ bending}$, $(N–H)_{in\text{-}plane\ bending}$, $(C–N)_{sp2\ stretching}$, $(NH_2)_{bending}$, $(C–C)_{aromatic\ stretching}$ and combinations thereof),[15–17] further underlining the conformational change. Accompanied by a slight shift of the concentration, these observations clearly denote the establishment of electronic interfacial interactions between NH_2-X and GO, probably involving π–π* stacking or hydrogen bonding. The same situation was encountered for the double frequency vibrational modes causing the vibronic A'_{00}, A'_{01} and A'_{02} features of the S_2 band, as well as for the higher frequency vibrational modes of the A''_{00} and A''_{01} features of the S_3 band.

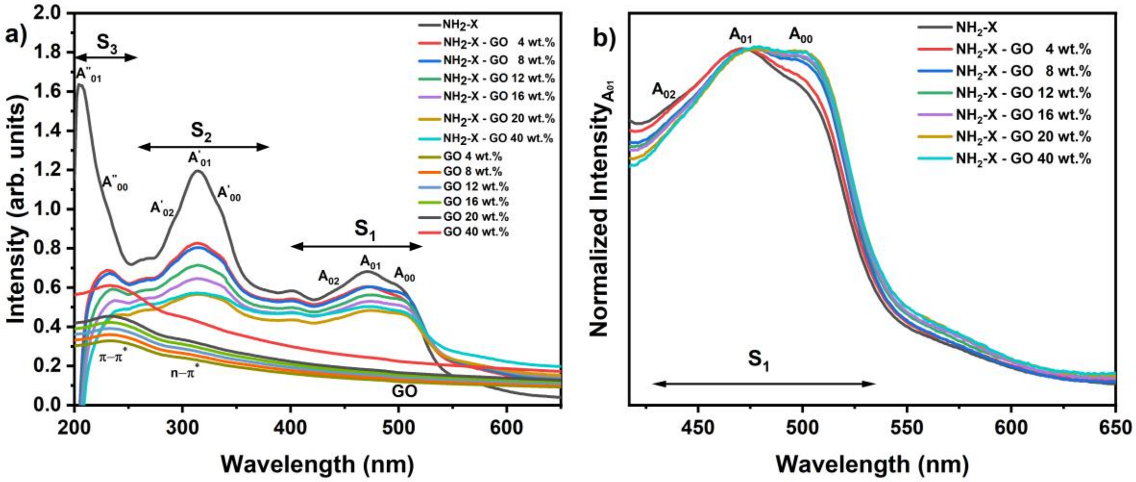

Figure S4. (a) Original UV-vis absorption spectra of NH_2-X and NH_2-X – GO hybrids at different concentrations. GO spectra were also included and correspond to the concentrations employed during the mixing process. (b) Normalized UV-vis absorption spectra of NH_2-X – GO (corrected for non-interacting GO absorption contribution) detailing the S_1 related absorption band with its vibronic structure and the effects of GO.

Section S4. UV-Vis spectra of GO

GO is understood as a highly defective π-conjugated 2D sheet, incorporating sp^3 domains from different types of oxygen functional groups (OFGs) at its surface and edges, while accompanied by major structural defects.[18] As such, it revealed a rather broad absorption spectrum exhibiting a π-π* transition around 230 nm and a weak n-π* transition at about 310 nm (Figure S5). These bands were superimposed on a rather featureless background, characteristic for a dense number of trap states fading towards lower absorption wavelengths, behaving like a heavily doped wide band-gap semiconductor. However, the rather flat background for wavelengths beyond 600 nm did not involve any electronic transitions but merely indicated scattering processes of the GO flakes, scaling with the GO concentration in solution.

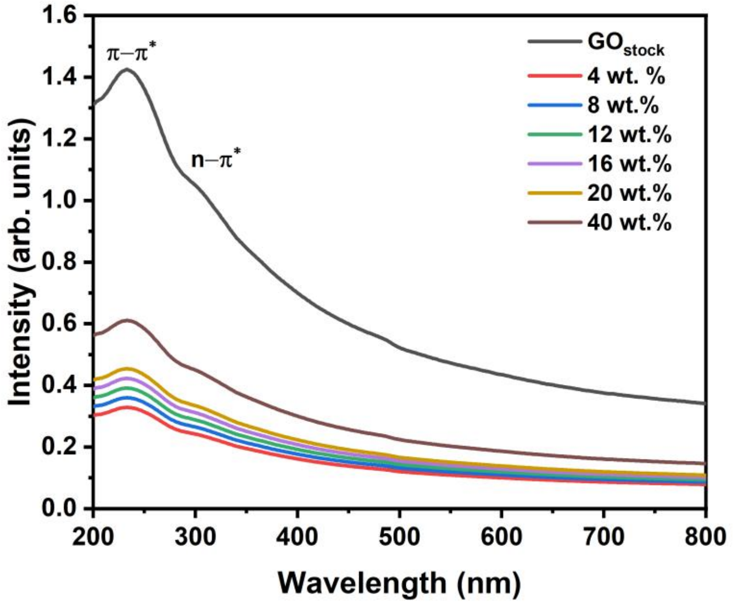

Figure S5. UV-Vis absorption spectrum of GO at initial concentration of the stock solution (0.2 mg/mL) and at diluted concentrations being employed in the mixing process.

Section S5. Photoluminescence emission spectra of NH_2-X – GO at 315 nm

Figure S6 shows the emission spectra of NH_2-X – GO charge transfer complexes using an excitation wavelength of 315 nm. Increasing GO content leads to a reduced fluorescence intensity, following a similar trend to that of analogous experiments carried out at an excitation wavelength of 480 nm (Figure 2c). While this clearly indicates that high energetic levels (S_2) contribute to populate the fluorescent-causing S_1 state in NH_2-X, with increasing GO concentration, this contribution becomes lower, thus confirming the electronic interface interactions according to the mechanism in Figure 3.

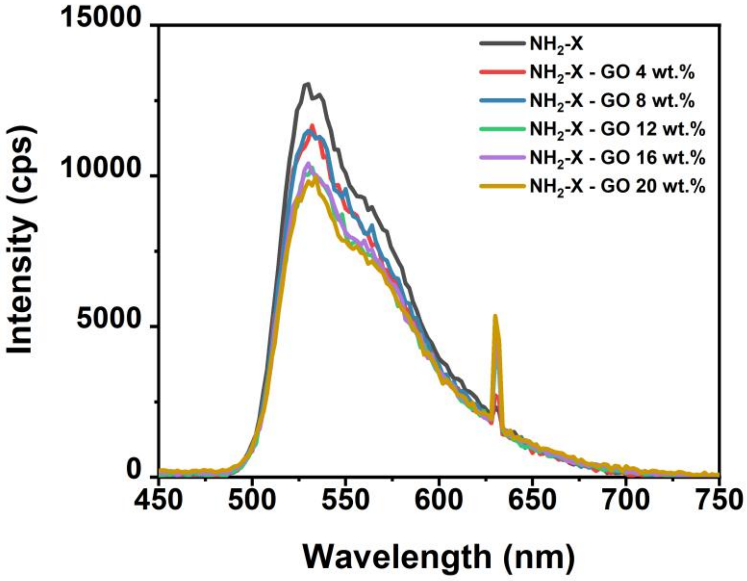

Figure S6. Photoluminescence emission spectra of NH_2-X – GO hybrids using an excitation wavelength of 315 nm.

Section S6. Fluorescence lifetime of NH_2-X and quenching mechanism

Steady-state photoluminescence spectra measurements were recorded with a Jobin-Yvon Horiba Fluorolog FL-3-11 using a Fluoromax phosphorimeter accessory containing a Horiba Jobin Ybon LED with a pulse duration <1.2 ns. The excitation wavelength was 450 nm and measurements were carried out using a 10^{-5} M of NH_2-X methanolic solution in quartz cuvettes. The fluorescence lifetime of the NH_2-X compound was found to be 2.9 ns (Figure S7a), while the NH_2-X – GO hybrid (0.4 %wt.) reveals 2.8 ns (Figure S7b).

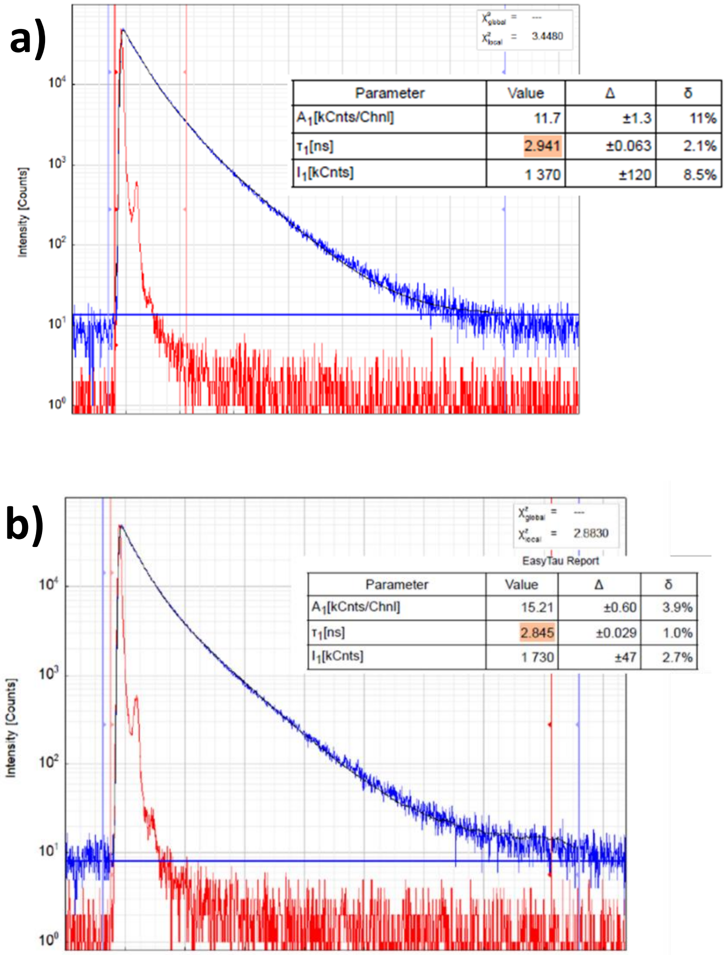

Figure S7. Fluorescence lifetime measurement of **a)** NH_2-X and **b)** NH_2-X – GO.

The measured fluorescence lifetime of the hybrid (2.8 ns) is essentially identical to that of pristine NH_2-X (2.9 ns), indicating no significant change in the intrinsic S_1 excited-state lifetime. This behavior is characteristic of static quenching, occurring through ground-state hybrid formation between NH_2-X and GO, being fully consistent with the linear Stern-Volmer behavior shown in **Figure 2d**.

The fluorescence quenching mechanism, was evaluated through the Stern-Volmer analysis (Equation S1),[19] which establishes a linear relationship between quencher concentration and decreased fluorescence intensity, according to **Equation S1**:

$$I_0/I = 1 + K_{SV} \cdot [GO] \qquad (\mathbf{S1})$$

where:

I_0: Fluorescence intensity of NH_2-X

I: Fluorescence intensity of the NH_2-X at a given concentration of GO

K_{SV}: Stern-Volmer constant

[GO]: graphene oxide concentration

Fluorescence quenching data were obtained by probing hybrid solutions prepared in a 10 mm quartz cuvette, leaving the NH_2-X concentration constant (0.025 mg/mL) upon increasing the amounts of GO by 0.002 mg/mL in each addition. Resulting I_0 / I values measured as a function of [GO], as represented in Figure 1d of main manuscript, can be fitted by a linear line, whose slope provides a Stern Volmer constant K_{SV} of 25.42 L/g.

According to **Equation S2**:

$$K_{SV} = \tau_0 \cdot k_q \qquad (\mathbf{S2})$$

where:

τ_0: Fluorescence lifetime of the NH_2-X

k_q: Quenching rate constant,

the k_q quenching rate constant can be calculated. With a measured fluorescence lifetime of 2.94 ns for NH_2-X (see Figure S6), a value of $8.65 \cdot 10^9$ L/g·s is obtained. More importantly, the form of the Stern-Volmer plot is indicative of the type of quenching mechanism (i. e., linear for static and tilted for dynamic). Thus, the linear fitting results from the experimental data shown in the Stern Volmer plot in Figure 2d, thus reveal that the fluorescence quenching of NH_2-X with GO occurs through a static quenching process, caused by the formation of physical contact between both components.[20–22] Furthermore, the calculated k_q constant of $8.65 \cdot 10^9$ L/g·s, falls well in the range of values representative for static quenching processes in xanthene-derived systems.[19,20]

Section S7. FE-SEM of TiO_2/NH_2-X – GO photoanodes

The prepared TiO_2/NH_2-X – GO photoanodes consistently reveal individual dark micrometer-sized islands randomly distributed on the surface of the TiO_2 films (Figure S8). The darker contrast, compared to TiO_2 surface, is indicative for enhanced conductivity, caused by the presence of conductive GO sheets (achieved after the electrochemical LSV reduction process). It should be noted that big chunks at the surface are due to the crystallization of Na_2SO_4 (employed electrolyte during the photoelectrochemical characterization).

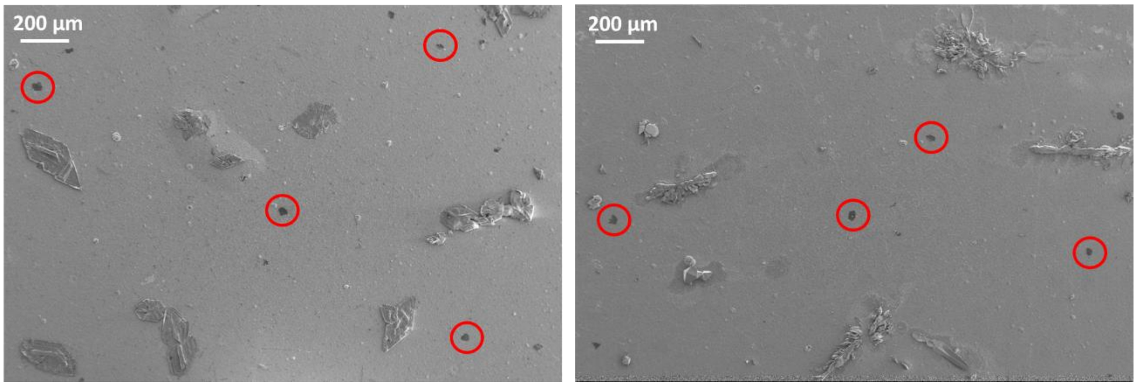

Figure S8. FE-SEM images of the TiO_2/NH_2-X – GO photoanodes.

Section S8. Preconditioning of the hybrid photoanodes

Prior to the photoelectrochemical measurements, the TiO_2/NH_2-X – GO photoanode underwent an electrochemical preconditioning treatment, consisting in a linear sweep voltammetry (LSV) step carried out from 0.4 V to –1.1 V under dark conditions and after that, the addition of sacrificial agent TEOA to the 0.1 M Na_2SO_4 followed by neutralization to pH 7 ($TEOA^+$) takes place. This kind of electrochemical reductive treatment are commonly employed to enhance the conductivity of GO, and thus its photoelectrical response, as it results in the irreversible reduction of oxygen functional groups and partial recovery of sp^2 conjugation of GO's graphene lattice.[23,24]

Figure S9 shows the results of the LSV pre-conditioning step. Here the photoanodes sensitized with the hybrid dye of highest GO concentration clearly reveal the important reduction peaks at – 0.65 V (vs Ag/AgCl), tentatively assigned to carbonyl and ester groups, and at – 0.9 V (vs. Ag/AgCl), may be ascribed to epoxy, hydroxyl, aldehydes and peroxides.[25] Lowering the GO concentration, decreases the corresponding response (overall those of basal plane groups at higher negative potentials), resulting in a current matching those of the photoanodes not containing GO.

The positive effects of the LSV preconditioning step can be clearly appreciated by comparison of Figure S10a and Figure S10b, which present photocurrent responses for the non-reduced and reduced photoanodes, respectively. The following trends are observed: (i) TiO_2 and TiO_2/NH_2-X photoanodes are not affected by the preconditioning step. (ii) Photocurrents for the non-reduced hybrid dye sensitized photoelectrodes show enhanced photocurrents, compared to bare TiO_2 photoelectrodes, reaching stable values of about 60 $\mu A/cm^2$ for a GO loading of 0.4 wt.%. However, the corresponding reduced photoanode even

reaches values of 110 μA/cm^2, being a consequence of GO's enhanced conductivity caused by LSV preconditioning step. (iii) Photoanodes sensitized with higher GO loadings show significantly lower or even no photocurrent enhancement effects upon the LSV. Moreover, it seems that increased amounts of GO loading in the hybrid, even negatively affect the stability of the photoanodes, most likely due to reactions with remaining functional groups. This points to the existence of an optimum amount of GO in the hybrid electrodes for achieving highest photocurrents and stability. Since this is offered by GO loadings of 0.4 wt.% in the TiO_2/NH_2-X – GO photoanodes, these constitute the system of choice for a more detailed photoelectrochemical characterization.

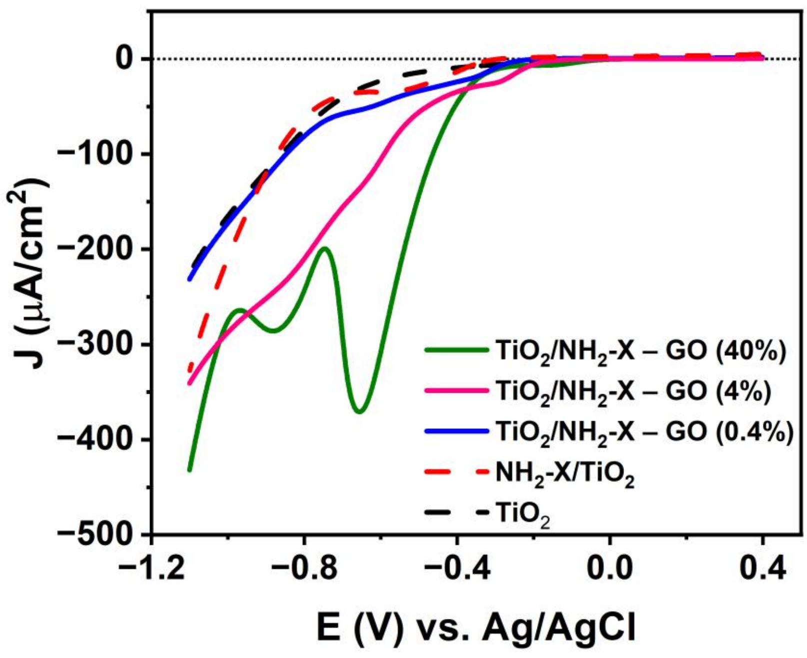

Figure S9. Linear sweep voltammetry (LSV) for the electrochemical reduction of GO in 0.1 M Na_2SO_4.

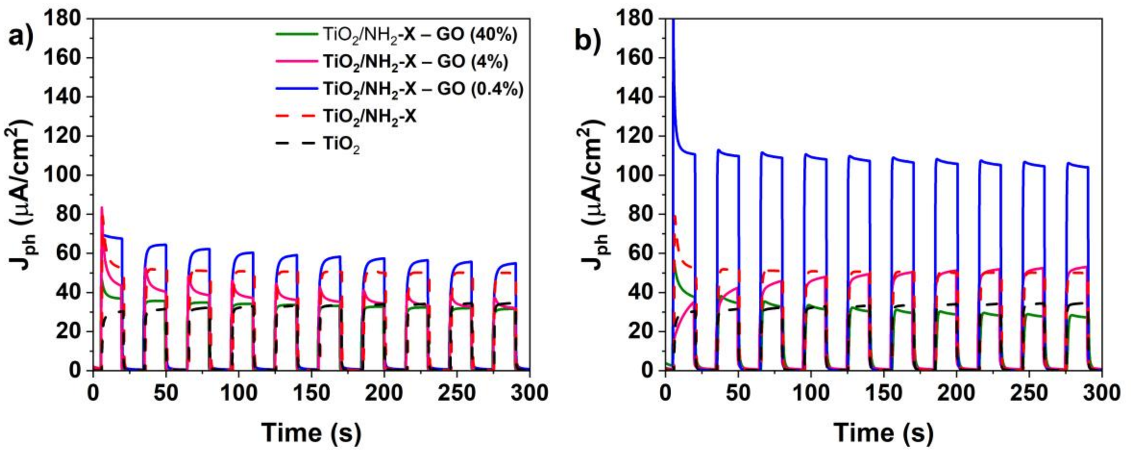

Figure S10. Photocurrent comparison at 0 V (vs. Ag/AgCl) of (a) non-reduced and (b) electrochemically reduced photoanodes. Electrolyte composed of 0.1 M Na_2SO_4 + 0.1 M $TEOA^+$. Irradiation conditions: 25 mW/cm^2.

Section S9. Effect of illumination conditions

To achieve dye stabilization under water splitting conditions, the illumination parameters should be carefully controlled in order to avoid photodegradation of dyes. In this sense, two types of experiments were carried out with the AM 1.5G filter: at 100 mW/cm^2 of light power intensity, which corresponds to standard illumination conditions, and at 70 mW/cm^2 adjusted by means of the solar lamp controller (Figures S11a and S11b, respectively).

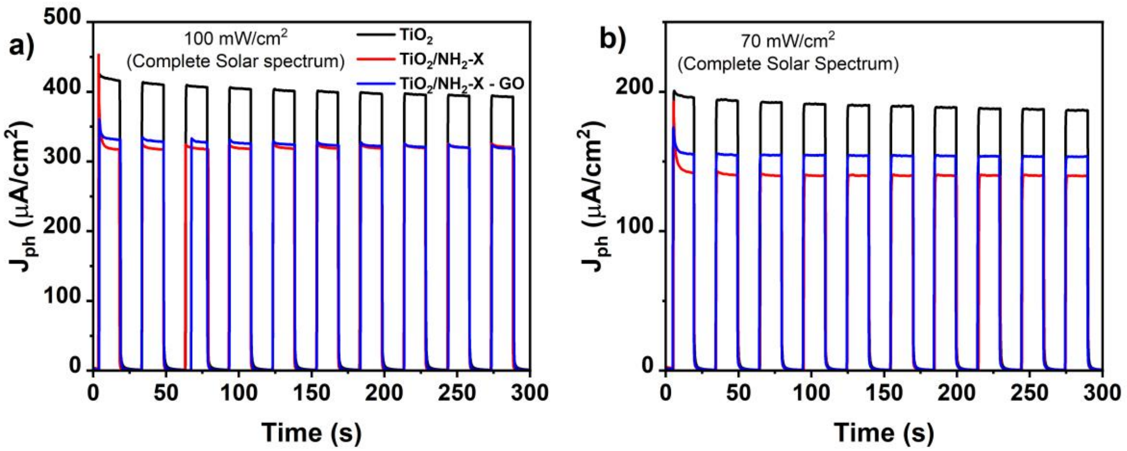

Figure S11. Transient photocurrent measurements at 0 V (vs. Ag/AgCl) using a light power intensity of (a) 100 mW/cm^2 and (b) 70 mW/cm^2. Note that the electrolyte employed during these experiments is composed of a 0.1M Na_2SO_4 + 0.1M $TEOA^+$ solution.

From these measurements, it can be observed that the sensitized systems (TiO_2/NH_2-X and TiO_2/NH_2-X – GO) are not stable under these illumination conditions, as photodegradation of NH_2-X is taking place. This finding was further corroborated when observing the aspect of photoanodes, which loss their characteristic orange color. Experiments with the cold mirror are shown in the main article, thus having a light intensity of 25 mW/cm^2.

Section S10. Stabilization of dye sensitized photoanodes

Working with dye sensitized photoanodes, commonly requires the establishment of some appropriate conditions. This firstly covers the use of adequate sacrificial electrolytes, preserving the stability of dye sensitized photoanodes, which suffer from photooxidation processes under photoelectrochemical water splitting operation conditions.[26] Figure S12 displays transient photocurrent measurements using different electrolytes. Experiment without sacrificial electrolyte show a clear presence of spikes upon illumination and a pronounced loss of photocurrent over time. A concomitant loss of the electrode initial orange color, thus indicates the poor stability of the dye-sensitized photoanodes under the applied electrochemical conditions. However, when $TEOA^+$ is used as sacrificial electrolyte while bringing the pH of the Na_2SO_4 electrolyte was brought to a value of 7, no spikes are encountered anymore and the photocurrent remains stable over time. This means that despite a possible protonation of NH_2-X while sensitizing the photoanode, no detrimental effect was found according to the aforementioned experimental findings. This situation thus provides suitable working conditions under which a meaningful set of photoelectrochemical measurements can be realized for the NH_2-X-dye sensitized TiO_2 photoanode.

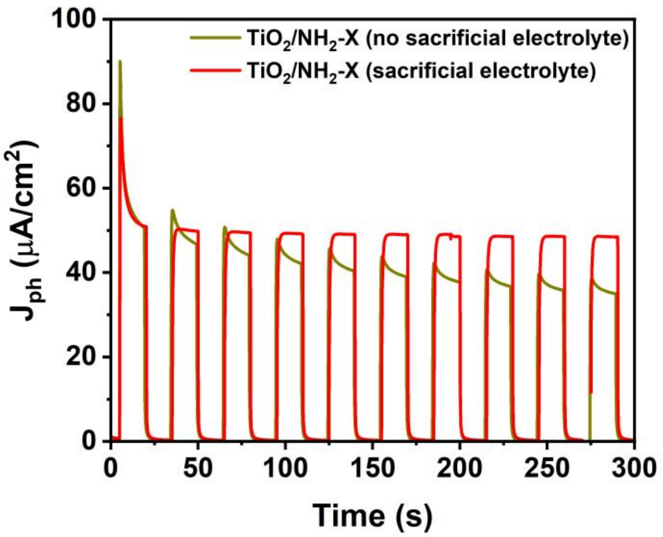

Figure S12. Transient on-off measurements of TiO_2/NH_2-X photoanodes using different electrolytic solutions. 0.1 M Na_2SO_4 was used in the "no sacrificial electrolyte", while 0.1 M Na_2SO_4 + 0.1 M $TEOA^+$ was employed in the "sacrificial electrolyte" measurements. Irradiation intensity: 25 mW/cm^2.

Section S11. Isolated role of GO in TiO_2 photoanodes

To evaluate the influence of isolated GO onto TiO_2 photoanodes, we carried out additional photoelectrochemical experiments shown in **Figure S13**. It can be observed that covering TiO_2 with a GO content equivalent to that of the GO – NH_2-X (40 %wt.) in the absence of NH_2-X leads to lower photocurrent than bare TiO_2. However, it should be mentioned that depending on the GO film thickness deposited onto TiO_2 photoelectrodes, GO can act either as hole blocking layer or hole transport layer.[27] As such, this strategy results in a different scenario, as no sensitizing component was involved.

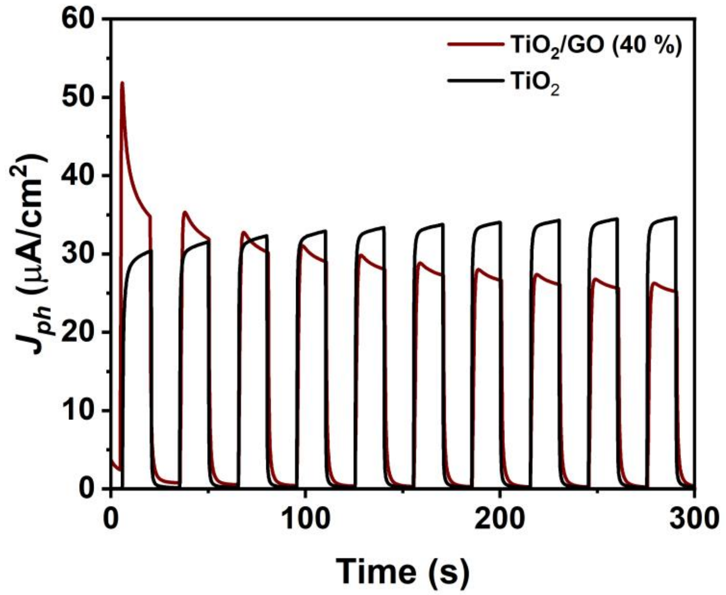

Figure S13. Effect of GO within a TiO_2 photoanode and its comparison with bare TiO_2.

References

(1) Garrido-Zoido, J. M.; Cajina, F.; Matamoros, E.; Gil, M. V.; Cintas, P.; Palacios, J. C. A Synthetically Benign One-Pot Construction of Enamino-Xanthene Dyes. *Org. Biomol. Chem.* **2022**, *20* (41), 8108–8119.

(2) Hummers, W. S.; Offeman, R. E. Preparation of Graphitic Oxide. *J. Am. Chem. Soc.* **1958**, *80*, 1339.

(3) Vallés, C.; David Núñez, J.; Benito, A. M.; Maser, W. K. Flexible Conductive Graphene Paper Obtained by Direct and Gentle Annealing of Graphene Oxide Paper. *Carbon* **2012**, *50* (3), 835–844.

(4) Núñez, J. D.; Benito, A. M.; Rouzière, S.; Launois, P.; Arenal, R.; Ajayan, P. M.; Maser, W. K. Graphene Oxide-Carbon Nanotube Hybrid Assemblies: Cooperatively Strengthened OH···O=C Hydrogen Bonds and the Removal of Chemisorbed Water. *Chem. Sci.* **2017**, *8* (7), 4987–4995.

(5) Rouzière, S.; Núñez, J. D.; Paineau, E.; Benito, A. M.; Maser, W. K.; Launois, P. Intercalated Water in Multi-Layered Graphene Oxide Paper: An X-Ray Scattering Study. *J. Appl. Crystallogr.* **2017**, *50* (3), 876–884. https://doi.org/10.1107/S1600576717006227.

(6) Rouzière, S.; Launois, P.; Benito, A. M.; Maser, W. K.; Paineau, E. Unravelling the Hydration Mechanism in a Multi-Layered Graphene Oxide Paper by in-Situ X-Ray Scattering. *Carbon* **2018**, *137*, 379–383.

(7) Gupta, A.; Chen, G.; Joshi, P.; Tadigadapa, S.; Eklund, P. C. Raman Scattering from High-Frequency Phonons in Supported n-Graphene Layer Films. *Nano Lett.* **2006**, *6* (12), 2667–2673.

(8) Ferrari, A. C.; Meyer, J. C.; Scardaci, V.; Casiraghi, C.; Lazzeri, M.; Mauri, F.; Piscanec, S.; Jiang, D.; Novoselov, K. S.; Roth, S.; Geim, A. K. Raman Spectrum of Graphene and Graphene Layers. *Phys. Rev. Lett.* **2006**, *97* (18).

(9) Ferrari, A. C.; Robertson, J. Interpretation of Raman Spectra of Disordered and Amorphous Carbon. *Phys Rev B* **2000**, *61* (20), 14095–14107.

(10) Zhang, C.; Dabbs, D. M.; Liu, L. M.; Aksay, I. A.; Car, R.; Selloni, A. Combined Effects of Functional Groups, Lattice Defects, and Edges in the Infrared Spectra of Graphene Oxide. *J. Phys. Chem. C* **2015**, *119* (32), 18167–18176.

(11) Acik, M.; Mattevi, C.; Gong, C.; Lee, G.; Cho, K.; Chhowalla, M.; Chabal, Y. J. The Role of Intercalated Water in Multilayered Graphene Oxide. *ACS Nano* **2010**, *4* (10), 5861–5868.

(12) Stankovich, S.; Dikin, D. A.; Piner, R. D.; Kohlhaas, K. A.; Kleinhammes, A.; Jia, Y.; Wu, Y.; Nguyen, S. B. T.; Ruoff, R. S. Synthesis of Graphene-Based Nanosheets via Chemical Reduction of Exfoliated Graphite Oxide. *Carbon* **2007**, *45* (7), 1558–1565.

(13) Colom, E.; Hernández-Ferrer, J.; Galán-González, A.; Ansón-Casaos, A.; Navarro-Rodríguez, M.; Palacios-Lidón, E.; Colchero, J.; Padilla, J.; Urbina, A.; Arenal, R.; Benito, A. M.; Maser, W. K. Graphene Oxide: Key to Efficient Charge Extraction and Suppression of Polaronic Transport in Hybrids with Poly (3-Hexylthiophene) Nanoparticles. *Chem. Mater.* **2023**, *35* (9), 3522–3531.

(14) Istif, E.; Hernández-Ferrer, J.; Urriolabeitia, E. P.; Stergiou, A.; Tagmatarchis, N.; Fratta, G.; Large, M. J.; Dalton, A. B.; Benito, A. M.; Maser, W. K. Conjugated Polymer Nanoparticle–Graphene Oxide Charge-Transfer Complexes. *Adv. Funct. Mater.* **2018**, *28* (23).

(15) Teixeira, A. M. R.; Santos, H. S.; Albuquerque, M. R. J. R.; Bandeira, P. N.; Rodrigues, A. S.; Silva, C. B.; Gusmão, G. O. M.; Freire, P. T. C.; Bento, R. R. F. Vibrational Spectroscopy of Xanthoxyline Crystals and DFT Calculations. *Brazil. J. Phys.* **2012**, *42* (3–4), 180–185.

(16) Watanabe, H.; Hayazawa, N.; Inouye, Y.; Kawata, S. DFT Vibrational Calculations of Rhodamine 6G Adsorbed on Silver: Analysis of Tip-Enhanced Raman Spectroscopy. *J. Phys. Chem. B* **2005**, *109* (11), 5012–5020.

(17) Wang, L.; Roitberg, A.; Meuse, C.; Gaigalas, A. K. Raman and FTIR Spectroscopies of Fluorescein in Solutions; *Spectrochim Acta A Mol Biomol Spectrosc*. **2001**; 57 (9), 1781-91.

(18) Dreyer, D. R.; Park, S.; Bielawski, C. W.; Ruoff, R. S. The Chemistry of Graphene Oxide. *Chem. Soc. Rev.* **2010**, *39* (1), 228–240.

(19) Wahba, M. E. K.; El-Enany, N.; Belal, F. Application of the Stern-Volmer Equation for Studying the Spectrofluorimetric Quenching Reaction of Eosin with Clindamycin Hydrochloride in Its Pure Form and Pharmaceutical Preparations. *Anal.Meth.* **2015**, *7* (24), 10445–10451.

(20) Kathiravan, A.; Anbazhagan, V.; Jhonsi, M. A.; Renganathan, R. Fluorescence Quenching of Xanthene Dyes by TiO2. *Z. Phys. Chem.* **2007**, *221* (7), 941–948.

(21) Povedailo, V. A.; Ronishenko, B. V.; Stepuro, V. I.; Tsybulsky, D. A.; Shmanai, V. V.; Yakovlev, D. L. Fluorescence Quenching of Dyes by Graphene Oxide. *J. Appl. Spectrosc.* **2018**, *85* (4), 605–610.

(22) Lakowicz, J. R. *Principles of Fluorescence Spectroscopy*; Springer, 2006.

(23) Zhou, A.; Bai, J.; Hong, W.; Bai, H. Electrochemically Reduced Graphene Oxide: Preparation, Composites, and Applications. *Carbon* **2022**, *191*, 301–332.

(24) Shao, Y.; Wang, J.; Engelhard, M.; Wang, C.; Lin, Y. Facile and Controllable Electrochemical Reduction of Graphene Oxide and Its Applications. *J. Mater. Chem.* **2010**, *20* (4), 743–748.

(25) Marrani, A. G.; Motta, A.; Schrebler, R.; Zanoni, R.; Dalchiele, E. A. Insights from Experiment and Theory into the Electrochemical Reduction Mechanism of Graphene Oxide. *Electrochim Acta* **2019**, *304*, 231–238.

(26) Ansón-Casaos, A.; Martínez-Barón, C.; Angoy-Benabarre, S.; Hernández-Ferrer, J.; Benito, A. M.; Maser, W. K.; Blesa, M. J. Stability of a Pyrimidine-Based Dye-Sensitized TiO2 Photoanode in Sacrificial Electrolytes. *J. Electroanal.Chem.***2023**, *929*, 117114.

(27) Hernández-Ferrer, J.; Ansón-Casaos, A.; Víctor-Román, S.; Sanahuja-Parejo, O.; Martínez, M. T.; Villacampa, B.; Benito, A. M.; Maser, W. K. Photoactivity Improvement of TiO2 Electrodes by Thin Hole Transport Layers of Reduced Graphene Oxide. *Electrochim Acta* **2019**, *298*, 279–287.
